# Supplementary material for: Genetically Corrected iPSC-Derived Neural Stem Cell Grafts Deliver Enzyme Replacement to Affect CNS Disease in Sanfilippo B Mice
Source: Mol Ther Methods Clin Dev. 2018 Jul 23;10:113–27. doi: 10.1016/j.omtm.2018.06.005 (PMC6076361; doi:10.1016/j.omtm.2018.06.005)
Supplement: Document S1. Figures S1–S3 [file mmc1.pdf]

OMTM, Volume *10*

Supplemental Information

Genetically Corrected iPSC-Derived Neural Stem Cell Grafts Deliver Enzyme Replacement to Affect CNS Disease in Sanfilippo B Mice

Don Clarke, Yewande Pearse, Shih-hsin Kan, Steven Q. Le, Valentina Sanghez, Jonathan D. Cooper, Patricia I. Dickson, and Michelina Iacovino

Supplementary Figure 1*. Biological Effect of NAGLU Treatment *in Vitro

Naglu$^{-/-}$ Neural Stem Cells (NSC) (A, B) or differentiated (C, D) cells treated for ten days with supernatant collected from Naglu overexpressing NSC (*N*-iNSCs) were compared to *Naglu*$^{+/+}$ untreated cells. (A) Nestin+ iNSCs, forward and side scatter (top) and LysoTracker staining (bottom). (B) FACS quantification: frequency of cell having bright LysoTracker signal (top) and mean LysoTracker signal (bottom). (C) Nestin- differentiated iNSCs, forward and side scatter (top) and LysoTracker staining (bottom). (D) FACS quantification: frequency cell having bright LysoTracker signal (top) and mean LysoTracker signal (bottom). Two-tailed *t*-test p-values .*$p < 0.05$, **$p < 0.01$, n = 3.

***Supplementary Figure 2*. Correction of Astrocytosis in *Naglu*$^{-/-}$ Mice Treated with Neural Stem Cells Overexpressing NAGLU (*N*-iNSCs) at Two Months**

(A) Schematic illustration intracerebroventricular (ICV, green) at bregma 1.54 mm; intraparenchymal (PAR, blue) at bregma -0.58 mm demonstrating regions of analysis (red squares), relative to the site of engraftment (ICV and PAR), including the motor cortex, striatum and ventral forebrain. (B) Representative bright-field images of half brain coronal sections of immunohistochemical staining of GFAP after *N*-iNSCs transplantation (-/- Treated) compared to unaffected heterozygous (Unaffected) and saline-injected controls (-/- Vehicle), scale bar = 1 mm. (C1-3) Representative immunohistological staining images for GFAP in higher magnification in the motor cortex (1), striatum (2) and ventral forebrain (3). Scale bar = 100 μm. Insert images taken

at higher magnification to highlight differences in morphology of GFAP-stained astrocytes, scale bar = 20 μm. Post-acquisition processing was applied to all images and included adjustments to brightness/contrast and Red Green Blue (RGB) curves using Adobe Photoshop CS6, to reduce improve visibility and consistency in colour tone. Area of GFAP immunoreactivity (μM^2) is summarized in the histograms. Values are shown as mean ± SEM (n = 3 mice per group), for p-values, see Table 1 and 2. *p < 0.05, **p < 0.01, ***p < 0.001.

Supplementary Figure 3. **Correction of Storage Material Accumulation in *Naglu*$^{-/-}$ mice Treated with Neural Stem Cells Overexpressing NAGLU (*N*-iNSCs) at Two Months**

(A) Schematic illustration intracerebroventricular (ICV, green) at bregma 1.54 mm; intraparenchymal (PAR, blue) at bregma -0.58 mm demonstrating regions of analysis (red squares), relative to the site of engraftment (ICV and PAR), including the motor cortex, striatum and ventral forebrain. (B) Representative bright-field images of half coronal sections immunostained for Lamp 1 in *Naglu*$^{-/-}$ mice (-/- Vehicle), heterozygotes (Unaffected) and *N*-iNSC transplanted (-/- Treated) mice. Scale bar = 1 mm. (C1-3) Representative bright-field images of the motor cortex (1), striatum (2) and ventral forebrain 9). Scale bar = 100 μm. Insert images taken at higher magnification to highlight cells with larger Lamp 1 staining lysosomes, scale bar = 20 μm. Post-acquisition processing was applied to all images and included adjustments to brightness/contrast and Red Green Blue (RGB) curves using Adobe Photoshop CS6, to reduce improve visibility and consistency in colour tone. Area of Lamp1 immunoreactivity (μM^2) is summarized in

the histograms showing differences between each treatment group. Values are shown as mean ± SEM (n = 3 mice per group) for p-values, see Table 1 and 2. *$p < 0.05$.

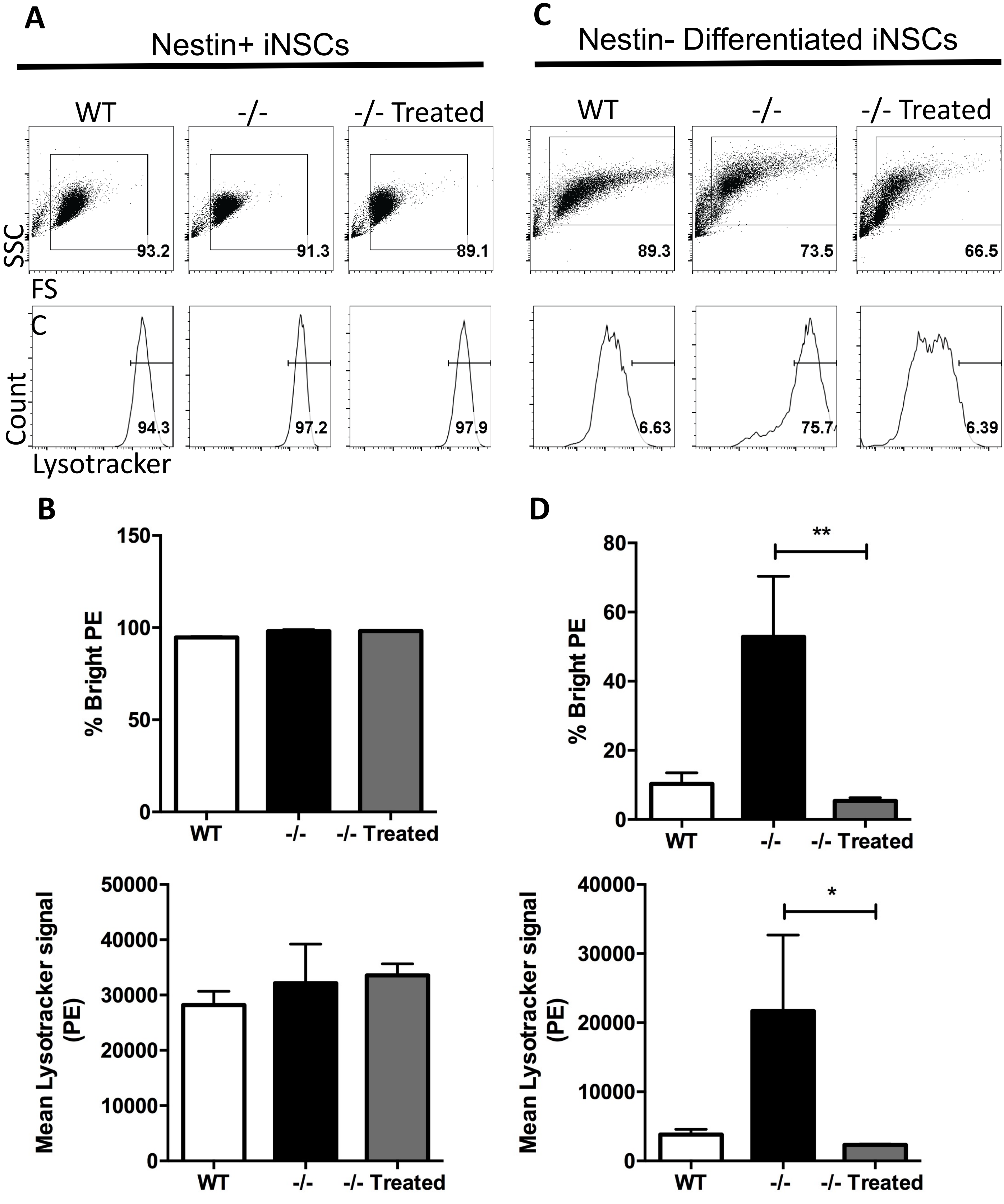
A
Nestin+ iNSCs
WT
-/-
-/- Treated
SSC
FS
93.2
91.3
89.1
C
Count
Lysotracker
94.3
97.2
97.9
C
Nestin- Differentiated iNSCs
WT
-/-
-/- Treated
89.3
73.5
66.5
6.63
75.7
6.39
B
% Bright PE
150
100
50
0
WT
-/-
-/- Treated
Mean Lysotracker signal (PE)
50000
40000
30000
20000
10000
0
WT
-/-
-/- Treated
D
% Bright PE
80
60
40
20
0
**
WT
-/-
-/- Treated
Mean Lysotracker signal (PE)
40000
30000
20000
10000
0
*
WT
-/-
-/- Treated

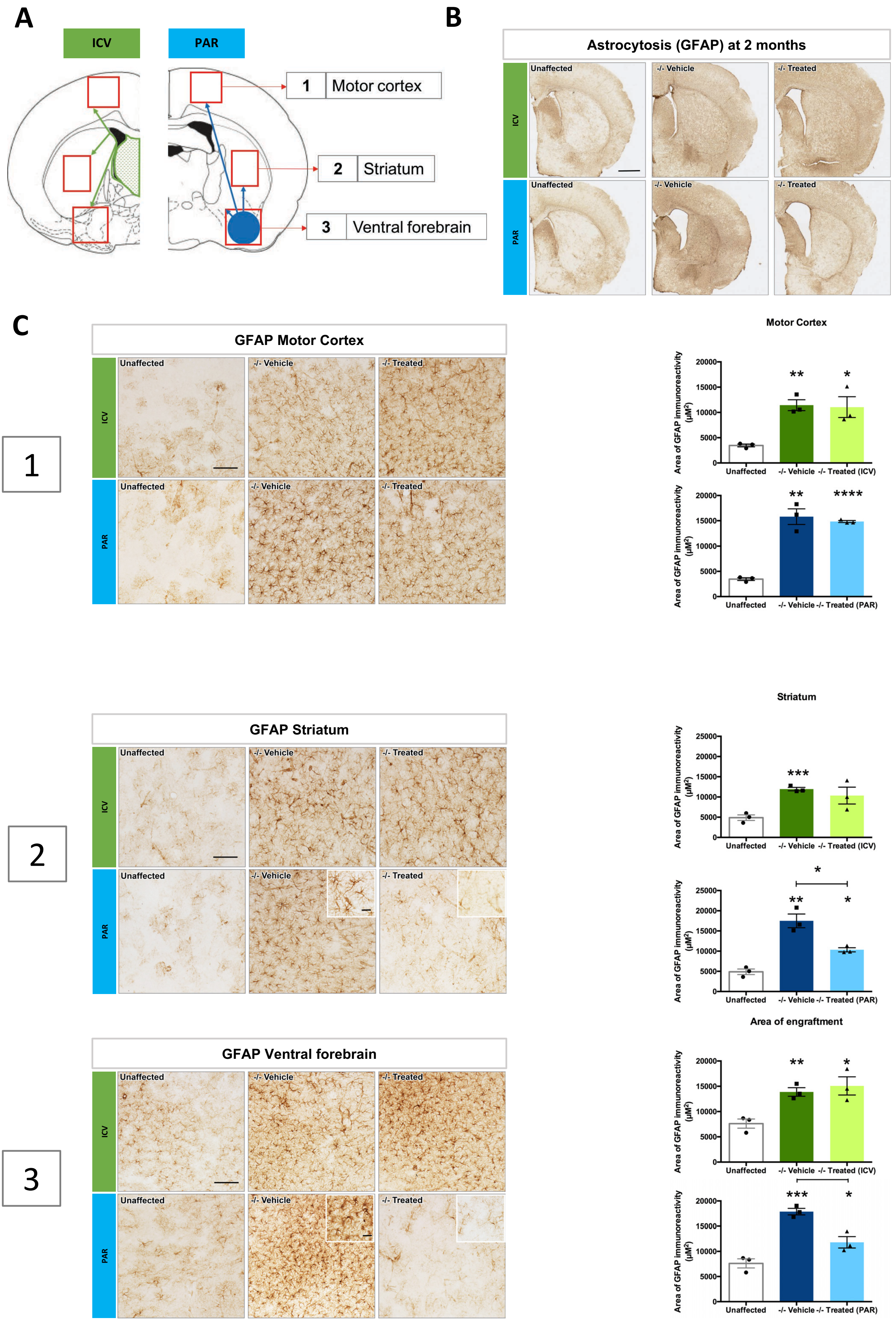

A
ICV
PAR
1 Motor cortex
2 Striatum
3 Ventral forebrain
B
Astrocytosis (GFAP) at 2 months
Unaffected
-/- Vehicle
-/- Treated
C
GFAP Motor Cortex
GFAP Striatum
GFAP Ventral forebrain
1
2
3
Motor Cortex
Striatum
Area of engraftment
Area of GFAP immunoreactivity (μM²)
Unaffected
-/- Vehicle
-/- Treated (ICV)
-/- Treated (PAR)

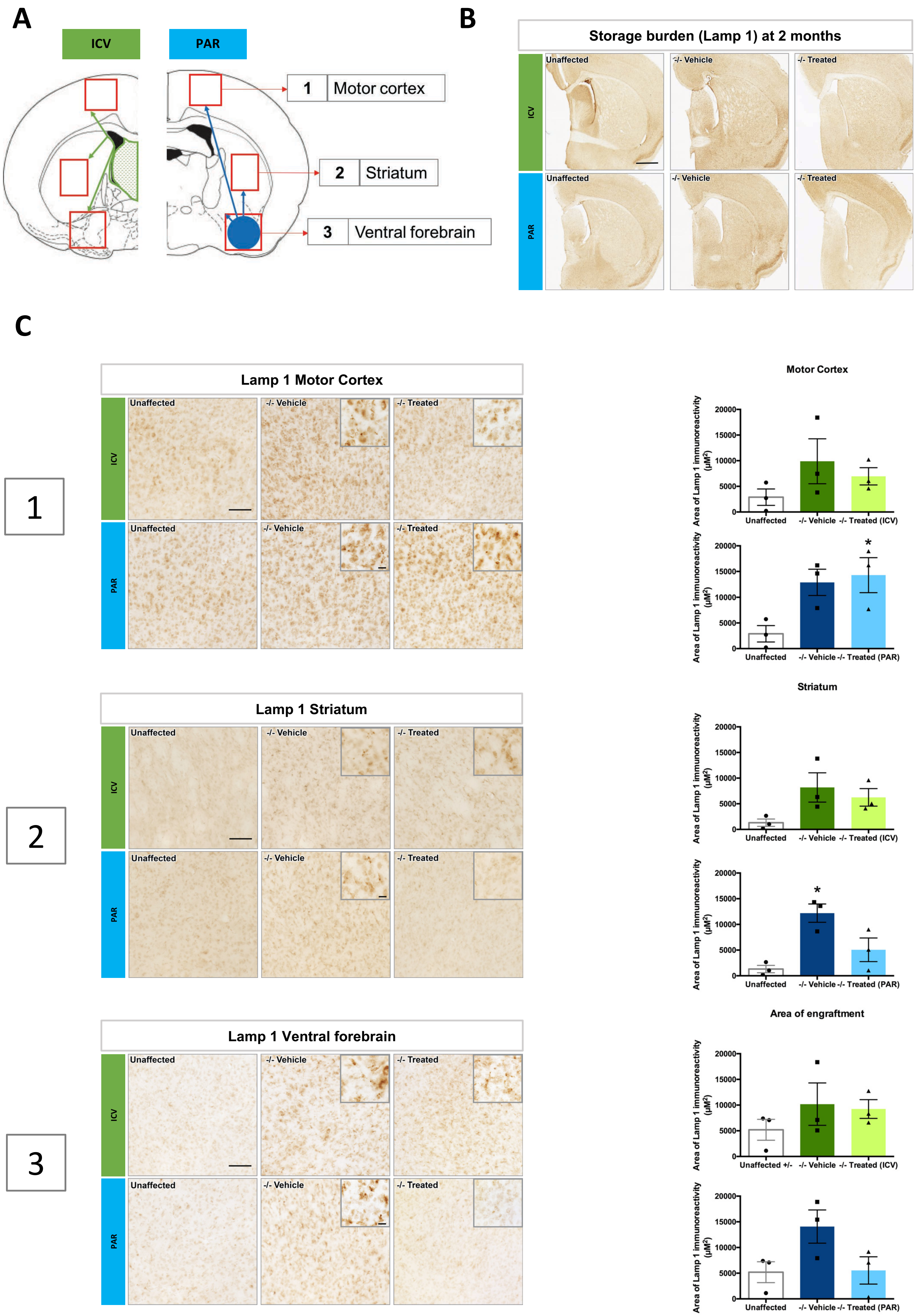

A
ICV
PAR
1 Motor cortex
2 Striatum
3 Ventral forebrain
B
Storage burden (Lamp 1) at 2 months
Unaffected
-/- Vehicle
-/- Treated
C
Lamp 1 Motor Cortex
Lamp 1 Striatum
Lamp 1 Ventral forebrain
1
2
3
Motor Cortex
Striatum
Area of engraftment
Area of Lamp 1 immunoreactivity (μM²)
Unaffected
-/- Vehicle
-/- Treated (ICV)
-/- Treated (PAR)
Unaffected +/-
