# Supplementary material for: Genetically Corrected iPSC-Derived Neural Stem Cell Grafts Deliver Enzyme Replacement to Affect CNS Disease in Sanfilippo B Mice
Source: Mol Ther Methods Clin Dev. 2018 Jul 23;10:113–27. doi: 10.1016/j.omtm.2018.06.005 (PMC6076361; doi:10.1016/j.omtm.2018.06.005)
Supplement: Document S2. Article plus Supplemental Information [file mmc2.pdf]

Molecular Therapy
Methods & Clinical Development
Original Article

Genetically Corrected iPSC-Derived Neural Stem Cell Grafts Deliver Enzyme Replacement to Affect CNS Disease in Sanfilippo B Mice

Don Clarke,[1,2,3] Yewande Pearse,[1,3] Shih-hsin Kan,[1] Steven Q. Le,[1] Valentina Sanghez,[1] Jonathan D. Cooper,[1] Patricia I. Dickson,[1] and Michelina Iacovino[1]

[1]Department of Pediatrics, Los Angeles Biomedical Research Institute at Harbor-UCLA Medical Center, Torrance, CA 90502, USA; [2]Phoenix Nest Inc., P.O. Box 150057, Brooklyn, NY 11215, USA

Sanfilippo syndrome type B (mucopolysaccharidosis type IIIB [MPS IIIB]) is a lysosomal storage disorder primarily affecting the brain that is caused by a deficiency in the enzyme α-*N*-acetylglucosaminidase (NAGLU), leading to intralysosomal accumulation of heparan sulfate. There are currently no treatments for this disorder. Here we report that, *ex vivo*, lentiviral correction of *Naglu*$^{-/-}$ neural stem cells derived from *Naglu*$^{-/-}$ mice (iNSCs) corrected their lysosomal pathology and allowed them to secrete a functional NAGLU enzyme that could be taken up by deficient cells. Following long-term transplantation of these corrected iNSCs into *Naglu*$^{-/-}$ mice, we detected NAGLU activity in the majority of engrafted animals. Successfully transplanted *Naglu*$^{-/-}$ mice showed a significant decrease in storage material, a reduction in astrocyte activation, and complete prevention of microglial activation within the area of engrafted cells and neighboring regions, with beneficial effects extending partway along the rostrocaudal axis of the brain. Our results demonstrate long-term engraftment of iNSCs in the brain that are capable of cross-correcting pathology in *Naglu*$^{-/-}$ mice. Our findings suggest that genetically engineered iNSCs could potentially be used to deliver enzymes and treat MPS IIIB.

INTRODUCTION

Mucopolysaccharidosis type IIIB (MPS IIIB), an inherited lysosomal disorder, is a sub-type of MPS III caused by deficiency of α-*N*-acetylglucosaminidase (EC 3.2.1.50 [NAGLU]), a lysosomal acid hydrolase that normally degrades heparan sulfate.[1] Heparan sulfate proteoglycans bind many ligands, modulate numerous cellular activities, and aid in tissue architecture and physiology.[2] Accumulation of heparan sulfate is associated with neurological dysfunction in MPS III, although the mechanism by which this may occur is not precisely known.

Children with MPS IIIB have severe neurological and behavioral deficits, generally leading to death in the first 20–30 years of life.[1] There is currently no effective treatment for MPS IIIB, and the clinical management of the disease is mainly symptomatic or palliative.[3] So far, research has focused on enzyme replacement therapy (ERT), gene therapy, hematopoietic stem cell transplantation, and substrate reduction to treat MPS IIIB, but these approaches have not been as successful as for other MPS diseases with less CNS involvement, largely because they have not been effectively targeted to the brain. For example, systemic ERT has been successful in treating some types of MPS with extensive somatic involvement (MPS I,[4] II,[5] IVA,[6] and VI[7]), but this approach is not feasible for MPS IIIB because of the inability of the NAGLU enzyme to cross the blood-brain barrier and alleviate the neurological deficits associated with MPS IIIB. Nonetheless, this problem could potentially be solved if the missing enzyme could be delivered to the brain via the cerebrospinal fluid (CSF), although life-long repeated administration would still be required.[8]

Gene therapy is an attractive tool for the treatment of monogenic disorders like MPS IIIB.[9–11] By delivering AAV9 vectors encoding NAGLU to the CSF of MPS IIIB mice, it was shown that the pattern of gene expression in the brain can be restored to that of healthy animals, giving rise to normal enzymatic activity. This results in the normalization of GAGs and Lamp 1 staining, a surrogate marker for lysosomal physiology, and resolution of the neuroimmune response together with the reversal of behavioral deficits and an extended lifespan.[10] Nevertheless, the promise of such gene therapy approaches has yet to be fulfilled clinically, and neural stem cells (NSCs) provide an alternative means to deliver enzymes to the brain.

Hematopoietic stem cell transplantation is currently available as a life-saving treatment for MPS I patients with CNS involvement but has not been effective for MPS IIIA,[12] presumably because of the low levels of either grafted cells or enzymes that reach the brain.[13] One possible solution would be direct implantation of NSCs into

Received 24 April 2018; accepted 7 June 2018;
https://doi.org/10.1016/j.omtm.2018.06.005.

[3]These authors contributed equally to this work.

Correspondence: Michelina Iacovino, Department of Pediatrics, Los Angeles Biomedical Research Institute at Harbor-UCLA Medical Center, Torrance, CA 90502, USA.
E-mail: miacovino@labiomed.org

This is an open access article under the CC BY license (http://creativecommons.org/licenses/by/4.0/).

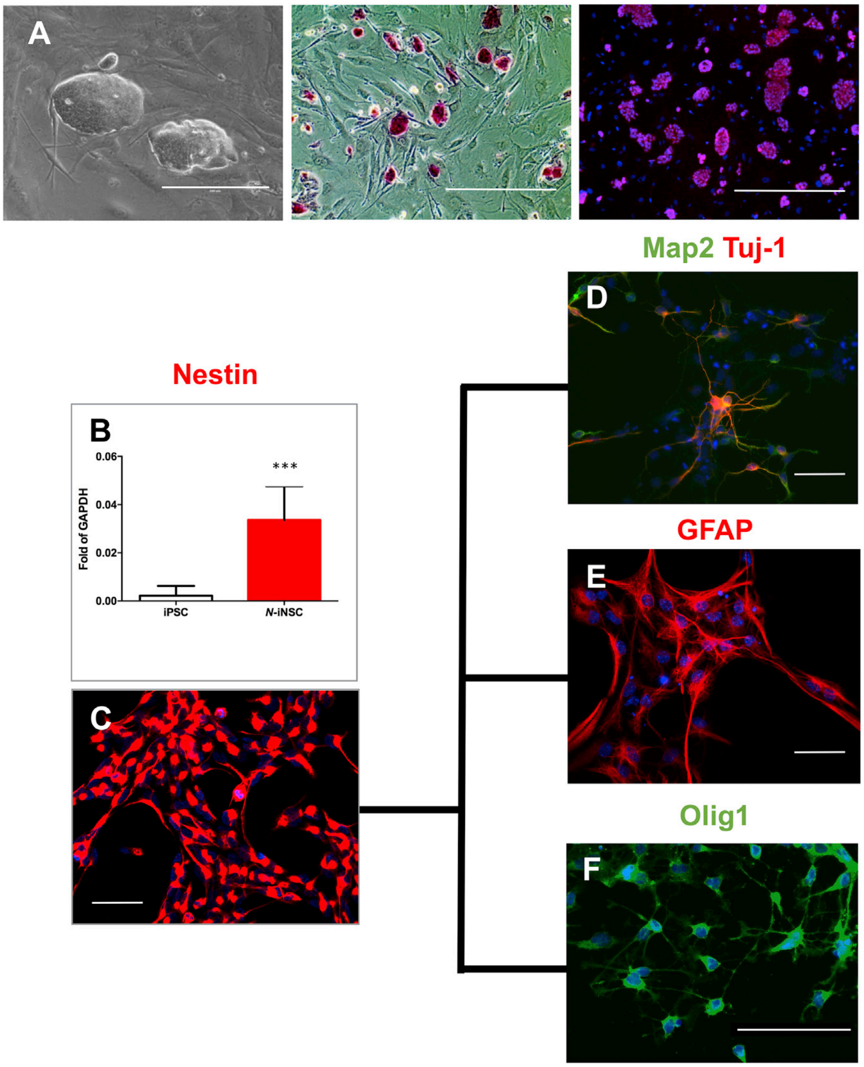

Figure 1. Characterization of Induced Pluripotent Stem Cells and Neural Stem Cells

(A–C) *Naglu*$^{-/-}$ iPSCs growing on feeder mouse embryonic fibroblasts (MEFs). (A) Phase contrast image, alkaline phosphatase staining, and Oct-4 (red) staining merged with nuclear staining (DAPI, blue). Scale bars, 200 μm (phase contrast) and 400 μm (alkaline phosphatase and Oct4). (B) Real-time qPCR probing *Nestin* gene expression after differentiation of iPSCs to neural stem cells (iNSCs; n = 5, p = 0.0005). Mean with SD. (C) Immunostaining of iNSCs with Nestin (red). Scale bar, 500 μm. (D–F) iNSCs were further differentiated into different cell types characterized by antibodies against Map2 and Tuj-1 (neuron markers) (D), GFAP (astrocyte marker) (scale bar represents 500 μm) (E), and Olig1 (oligodendrocyte marker) (scale bars represent 100 μm) (F). Nuclear staining with DAPI (blue) was overlaid in (C)–(F). Post-acquisition processing included adjustment of brightness and contrast in (C)–(F) using Adobe Photoshop CS6 to reduce over-exposure. ***p < 0.001.

the brain to provide a relatively long-term supply of secreted enzymes to the CNS.[14] Such NSCs can engraft and reduce lysosomal storage in MPS VII mice[15,16] and other lysosomal storage disorders.[17] Most studies have used NSC lines isolated from various sources, but another possibility is the development of induced pluripotent stem cells (iPSCs) to generate NSCs. The benefit of this approach is that gene-corrected NSCs could be developed from patient-derived iPSCs, providing a more optimal graft for transplantation by avoiding the issue of immune rejection.

Here we reprogrammed *Naglu*$^{-/-}$ mouse embryonic fibroblasts (MEFs) into iPSCs and corrected them *ex vivo* using a lentiviral vector to drive human *NAGLU* overexpression. We showed that corrected NSCs derived from these iPSCs expressed and secreted functional NAGLU *in vitro* and corrected their lysosomal defects. Upon intracranial transplantation of these corrected NSCs into *Naglu*$^{-/-}$ mice from birth, NAGLU activity was partially restored. Engrafted cells produced a marked reduction in lysosomal pathology and astrocytosis and reduced microglial activation to normal levels. These data also provide a promising proof of principle for this approach and pave the way to performing similar approaches to treat MPS IIIB patients with their own derived and corrected iPSCs.

RESULTS

Characterization of iPSCs and NSC Derivation and Differentiation

Naglu$^{-/-}$ MEFs were reprogrammed to iPSCs using lentiviral transduction of OCT4, KLF4, SOX2, and c-MYC.[18] Morphology, alkaline phosphatase staining, and immunofluorescence for Oct4 were used to confirm their pluripotency (Figure 1A). iPSC clones were differentiated to NSCs (iNSCs) as described previously,[19] and Nestin (an NSC marker) was expressed at the mRNA and protein levels (Figures 1B and 1C). The iNSCs were further differentiated into neurons, astrocytes, and oligodendrocytes to show their neural and glial lineage potential (Figures 1D–1F). These differentiated cells showed an appropriate cell type-specific morphology and stained positively for the phenotypic markers Tuj1 and Map2, glial fibrillary acidic protein (GFAP), and Olig1, respectively. These results demonstrate that *Naglu*$^{-/-}$ MEFs can not only be successfully reprogrammed to iPSCs but were also capable of differentiating into neurons and glia of the appropriate lineage.

NAGLU Gene Therapy and Subsequent Biochemical Characterization

To correct the MPS IIIB-associated genetic defects, we overexpressed full-length human *NAGLU* cDNA in *Naglu*$^{-/-}$ iNSCs using lentiviral

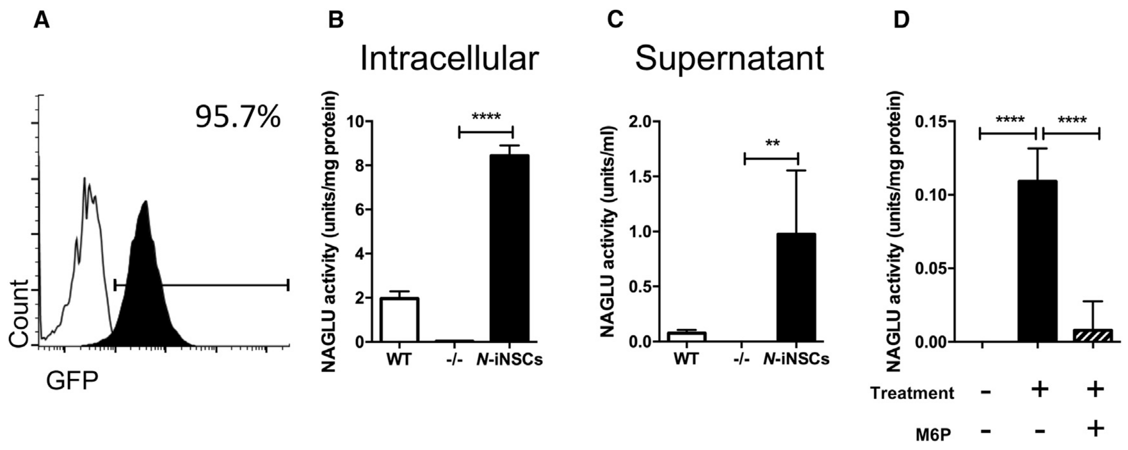

Figure 2. NAGLU Gene Therapy and Subsequent Biochemical Characterization

(A) FACS analysis of *Naglu*$^{-/-}$ iNSCs (white) transduced with *NAGLU-GFP* (black). (B and C) Quantifying secreted (C) and intracellular (B) NAGLU enzyme activity in iNSCs overexpressing NAGLU (*N*-iNSCs). Student's t test, $p < 0.0001$ and $p = 0.0047$, respectively. (D) Cellular uptake and inhibition assay on iNSCs (*Naglu*$^{-/-}$) treated with supernatant collected from *N*-iNSCs in the presence or absence of 5 mM mannose-6-phosphate (M6P). n = 4, one-way ANOVA, $p < 0.0001$. (B–D) Mean with SD. Intracellular NAGLU activity is presented as units per milligram of protein, whereas the secreted NAGLU activity is presented as units per milliliter expressed as mean ± SD, where a unit is defined as the release of 1 nmol of 4-MU per hour at 37°C. **$p < 0.01$, ****$p < 0.0001$.

transduction. The viral construct also carried a GFP reporter, and, following transduction, the cells were sorted using fluorescence-activated cell sorting (FACS) to ensure pure, unsilenced expression of NAGLU (Figure 2A). We refer to corrected cells as *N*-iNSCs. *N*-iNSCs had intracellular NAGLU activity 4-fold greater than the wild-type (WT) and secreted NAGLU at a level 14-fold greater than the WT (Figures 2B and 2C).

Previous attempts to produce recombinant NAGLU have resulted in a poorly phosphorylated enzyme that demonstrates little to no intracellular uptake.[20–22] To determine whether the NAGLU secreted from our *N*-iNSCs was able to enter *Naglu*$^{-/-}$ cells in a mannose-6-phosphate (M6P)-dependent manner, we performed cellular uptake assays with an excess amount of M6P to inhibit NAGLU uptake via M6P receptors (M6PRs). We harvested supernatant from *N*-iNSCs and applied it to *Naglu*$^{-/-}$ iNSCs in the presence or absence of 5 mM M6P. After 4 hr of treatment, NAGLU activity reached a mean of 6.5% ± 1.15% of WT levels. Intracellular uptake was completely inhibited in the presence of 5 mM M6P (Figure 2D). These data provide evidence that *N*-iNSCs secreted an appropriately phosphorylated NAGLU that is capable of cross-correcting *Naglu*$^{-/-}$ cells via M6PRs.

A hallmark of MPS IIIB disease in patients and also in *Naglu*$^{-/-}$ mice is enlarged lysosomes. To quantify the effect of NAGLU secreted from *N*-iNSCs on lysosomal size, we used LysoTracker, a fluorescent acidotropic probe for labeling and tracking acidic organelles in live cells.[23] We measured the frequency and mean intensity of the signal in LysoTracker-positive cells using FACS.[24] We speculated that *Naglu*$^{-/-}$ mouse-derived iNSCs would have larger lysosomes compared with WT NSCs and that, upon treatment with NAGLU-containing supernatant, the size of their lysosomes would return to WT level. However, we did not detect significant differences in the frequency of *Naglu*$^{-/-}$ iNSCs with a high LysoTracker signal compared with WT NSCs or any changes in their mean LysoTracker signal intensity (Figures S1A and S1B). Because autophagy is upregulated in NSCs to fulfil their high energy demand,[25] and because this process requires membrane fusion to lysosomes,[26] an altered LysoTracker signal would be more readily detected in differentiated NSCs, and this might reveal differences between iNSCs of different genotypes. Therefore, we differentiated NSCs into neuronal and glial lineages (NSC Nestin−) and measured their LysoTracker signal. When the iNCSs were differentiated, the frequency of cells with a higher LysoTracker signal was greater in *Naglu*$^{-/-}$ cells compared with WT cells. Upon treatment with conditioned medium from our lentivirally corrected *N*-iNSC cells, *Naglu*$^{-/-}$ cells showed a 63% decrease in the frequency of cells with a high LysoTracker signal and a 10-fold decreased mean LysoTracker signal intensity (Figures S1C and S1D).

NSCs Engraftment

We next grafted *N*-iNSCs into newborn *Naglu*$^{-/-}$ mice to assess their distribution and the subsequent effect of secreted NAGLU on disease-associated neuropathology *in vivo*. We independently used two different routes to deliver *N*-iNSCs; they were either injected bilaterally into the lateral ventricles (intracerebroventricular [ICV]) or the striatum (intraparenchymal; PAR in Figure 3A). Two months after transplantation, we examined the forebrain for evidence of *N*-iNSC engraftment. The left hemisphere was fixed and cut into 40-μm coronal sections for GFP immunohistochemistry (Figure 3B). The right hemisphere was used for evaluation of GFP expression using flow cytometry analysis (FACS) and NAGLU enzyme activity for both ICV and intraparenchymal injections (Figures 3C and 3D).

GFP-positive cells were detected in five of six ICV-grafted animals, as measured by FACS (Figure 3C). 1%–5% of residual enzyme activity is

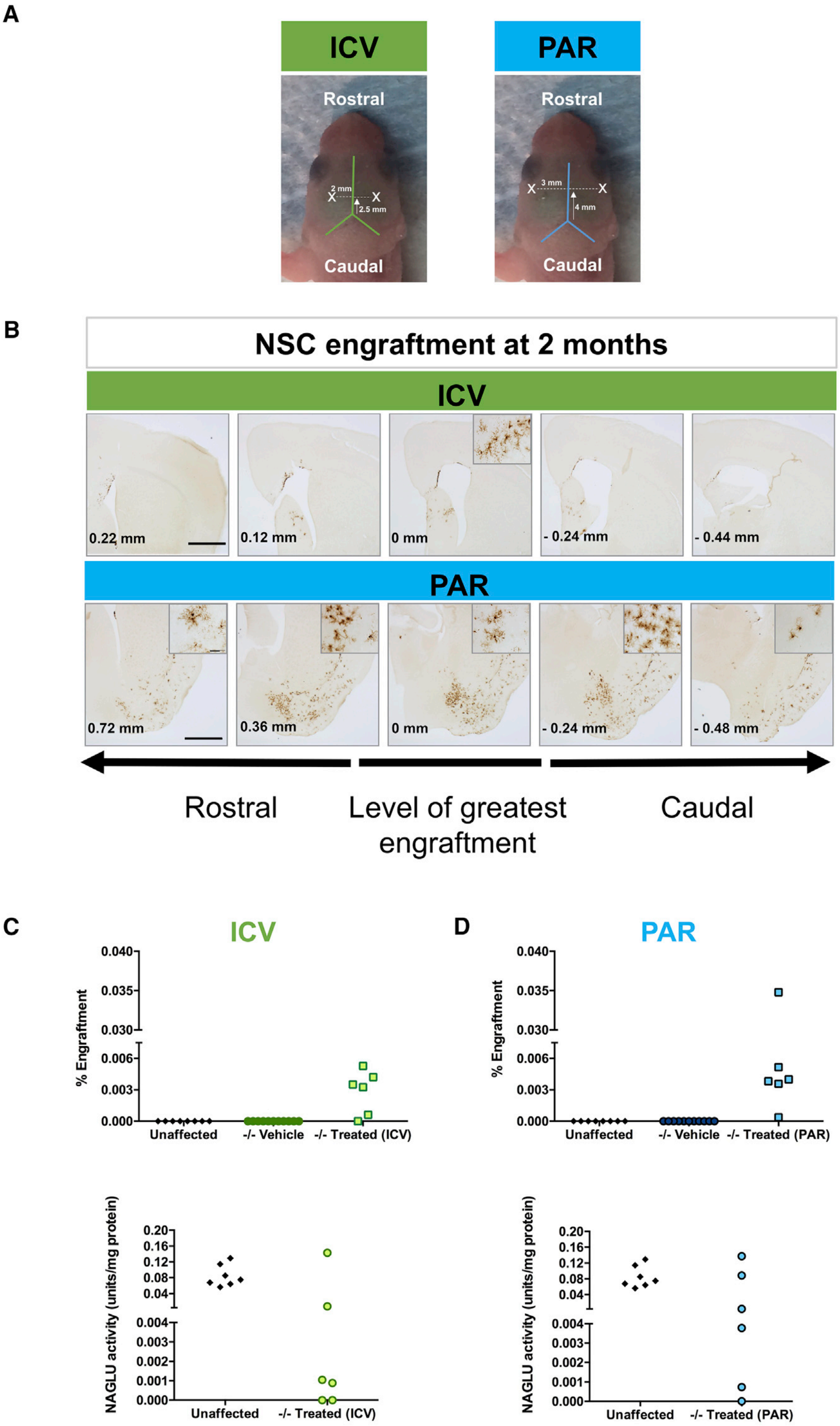

Figure 3. NAGLU Corrected *N*-iNSC Grafting after 2 Months

(A) Annotated image of the parameters for intracerebroventricular (ICV, green) and intraparenchymal (PAR, blue) injections. X indicates represented injection sites. (B) Immunohistological staining of 40-μm representative sections for GFP, shown in low magnification, following ICV and intraparenchymal transplantion of *N*-iNSCs at birth (cells have a GFP reporter) in *Naglu*$^{-/-}$ mice 2 months after transplantation. Post-acquisition processing included adjustments to brightness and contrast and red green blue (RGB) curves using Adobe Photoshop CS6 to improve visibility and consistency in color tone. Scale bar, 1 mm. Rostrocaudal distribution of *N*-iNSCs relative to the level of greatest engraftment is shown in millimeters (bottom left corner). FACS analysis showing percentage engraftment (C and D, top) and NAGLU enzyme activity assays are shown in units per milligram of protein (C and D, bottom), performed on brain lysates following ICV and intraparenchymal injection (n = 6 in each treatment).

typically considered sufficient to correct storage accumulation in lysosomal storage disorders (LSDs).[27,28] NAGLU activity levels in two of six animals were greater than 5% of the level present in unaffected controls (Figure 3D). Similarly, FACS analysis showed that five of six intraparenchymally grafted animals possessed GFP-positive cells, but four of six transplanted animals had NAGLU activity greater than 5% of unaffected animals. When injected via ICV, NSCs need to cross the ependyma to spread into the parenchyma, posing a challenge for the cells to efficiently graft, resulting in a lower number of grafted animals using this route.

To determine where the cells had engrafted, we immunostained the brain sections to identify GFP-positive cells. Figure 3B shows the relative amount and distribution of *N*-iNSC engraftment in ICV and intraparenchymally grafted brains. Relatively few cells were detected in the parenchyma of ICV grafted brains, with a limited rostrocaudal spread of no more than 0.5 mm in either direction from the injection site. The majority of GFP-positive cells in these ICV-grafted brains were located within the lateral septum and the dorsal border of the lateral ventricle, with fewer cells within and above the *corpus callosum* in the motor cortex (Figure 3B).

In contrast, *N*-iNSC engraftment was markedly greater in the brains of intraparenchymally grafted animals, with GFP-positive cells present over a greater rostrocaudal distance of at least 1.5 mm away from the site of injection. The majority of these GFP-positive cells were within the ventral and ventrolateral forebrain, within the *globus pallidus*, ventral pallidum, and piriform cortex, extending caudally into nuclei of the amygdala, as well as the ventral and lateral striatum itself (Figure 3B). Fewer GFP-positive cells could also be detected along the fiber tracts of

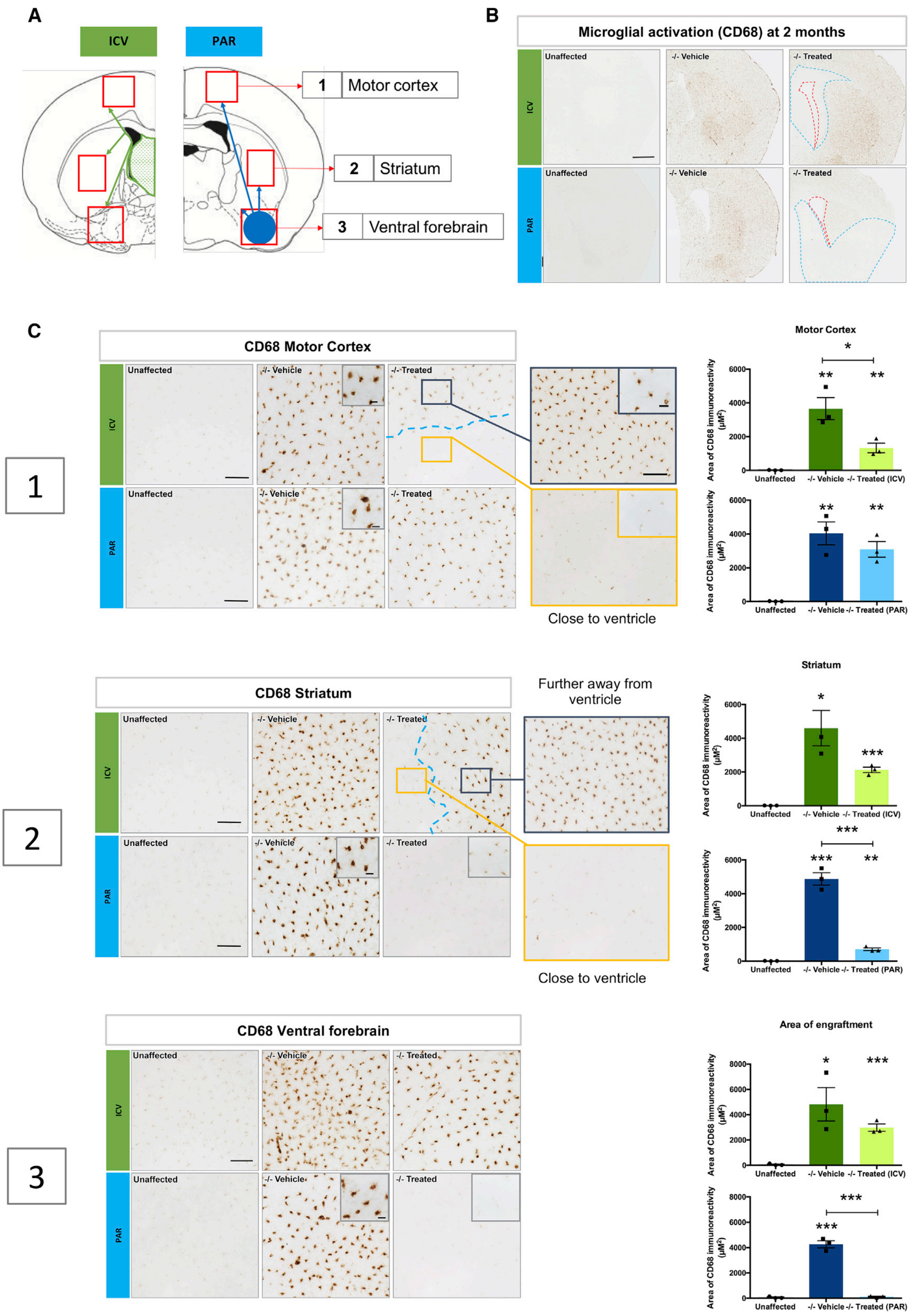

(legend on next page)

the *corpus callosum* separating the striatum from the cerebral cortex (Figure 3B). In addition, a small number of cells was detected in and around the hippocampal formation (Figure 3B), with no preference for any particular subfield.

Effect of *N*-iNSCs upon Microglial Activation

A neuroimmune response is a common feature of MPS IIIB,[29–31] and *Naglu*$^{-/-}$ mice show pronounced microglial activation.[11,30,31] To assess whether *N*-iNSCs had any effect upon microglial activation, we stained sections of unaffected control (*Naglu*$^{+/-}$), vehicle-injected *Naglu*$^{-/-}$ (−/− vehicle), and iNSC-grafted *Naglu*$^{-/-}$ (−/− treated) brains for the microglial marker CD68. Thresholding image analysis was then used to quantify the effect of *N*-iNSCs upon CD68 immunostaining both close to the site of maximum engraftment (local effects of grafts, within the motor cortex and dorsal striatum in ICV mice, and within the ventral forebrain in intraparenchymally grafted mice) and further away (within the ventral striatum) in ICV-grafted mice and in the motor cortex in intraparenchymally grafted mice to assess the potential effect of secreted enzyme to cross-correct pathology (Figure 4A).

As expected, there was significantly more CD68 immunoreactivity in vehicle-injected *Naglu*$^{-/-}$ mice compared with unaffected control mice, and this could be seen in widespread brain regions (Figures 4B and 4C). At high magnification, these microglia were darkly stained and rounded in shape, typical of activated microglia (Figure 4C, 1–3).

For both ICV and intraparenchymally grafted brains, graded effects on the distribution of CD68 immunoreactivity were observed (Figure 4B), with much less staining for this marker and more pronounced effects on microglial morphology closer to the sites of engraftment. In the ICV-grafted brains, where most of the *N*-iNSC cells were found in the septum, CD68 staining was markedly reduced in the surrounding regions (Figure 4B) but remained more intense farther away from these sites, revealing what appeared to be a boundary beyond which grafts did not influence microglial activation to the same extent. Indeed, these effects were evident both dorsally in the motor cortex and in the striatum, suggesting a therapeutic effect extending in both directions from the site of engraftment. Close to the ventricles, where the greatest number of cells engrafted in ICV mice, the level of CD68 staining resembled that of the unaffected brains (Figure 4C, 1), and thresholding image analysis showed this effect of ICV grafts to be significant, whereas, farther away from the ventricle, CD68 immunoreactivity remained quantitatively at a level similar to vehicle-injected *Naglu*$^{-/-}$ mice, albeit appearing morphologically less activated (Figure 4C, 1). Farther away in the striatum of ICV-grafted mice, the level of CD68 immunostaining was partly reduced compared with untreated *Naglu*$^{-/-}$mice by approximately half, but this was not significant statistically ($p = 0.0808$; Figure 4C, 2). When CD68 immunoreactivity was analyzed within the ventral forebrain of ICV-grafted animals (at the greatest distance from where the engrafted cells were detected), we saw an even smaller reduction in CD68 immunoreactivity compared with the striatum and motor cortex (Figure 4C, 3).

A similar overall reduction in microglial activation was observed in intraparenchymally grafted animals compared with vehicle-injected *Naglu*$^{-/-}$ mice (Figure 4B). Within the motor cortex of the intraparenchymally grafted animals (at the greatest distance from where most of the *N*-iNSC cells had engrafted), we observed a 1.3-fold reduction in CD68 immunoreactivity and fewer large intensely stained ameba-shaped microglia compared with vehicle-injected *Naglu*$^{-/-}$ mice (Figure 4C, 1). In contrast, when we analyzed the striatum and the ventral forebrain of intraparenchymally grafted animals versus vehicle-injected controls, we observed highly significant 7-fold ($p = 0.0004$) and 35-fold ($p = 0.0001$) reductions in CD68 immunoreactivity, respectively. In these regions, CD68 staining was no different than the unaffected mice, and these CD68-positive microglia had a highly ramified morphology and small cell body typical of resting or less activated microglia (Figure 4C, 2 and 3).

Effect of *N*-iNSC on Astrocytosis

Another common feature of the innate immune response in MPS IIIB is astrocytosis,[31] which can be revealed by GFAP immunostaining. The level of GFAP immunostaining in vehicle-treated *Naglu*$^{-/-}$ mice ranged between regions from 1.5- to 4-fold higher than unaffected mice among the motor cortex, striatum, and ventral forebrain (Figures S2A and S2B) and was highly significant regardless of iNSC delivery route (Figures S2B and S2C, 1–3). A readily discernable zone of reduced GFAP immunostaining was evident in the region of grafted cells in intraparenchymally but not ICV-grafted brains (Figure S2B). In these ICV-grafted animals, no difference in GFAP immunoreactivity was apparent in *N*-iNSC-treated animals

Figure 4. Reduction of Microglial Activation in Naglu$^{-/-}$ Mice Treated with Neural Stem Cells Overexpressing NAGLU

(A) Schematic illustration of ICV injection (green) at the bregma (1.54 mm) and PAR injection (blue) at the bregma (−0.58 mm), demonstrating regions of analysis (red squares) relative to the site of engraftment (ICV and PAR), including the motor cortex, striatum, and ventral forebrain. (B) Representative bright-field images taken at a low magnification of half-brain coronal sections of immunohistochemical staining of CD68 in a mouse injected with *N*-iNSCs (−/− treated) compared with unaffected heterozygous (unaffected) and vehicle-injected *Naglu*$^{-/-}$ mice (−/− vehicle). Scale bar, 1 mm. The locations of ICV and intraparenchymal injections are outlined by red dotted lines. The blue dotted lines outline regions where CD68 staining is less pronounced, indicating a reduction of microglial activation. (C) Representative bright-field images of the motor cortex (1), striatum (2), and ventral forebrain (3) of immunohistological staining for CD68 in −/− vehicle compared with unaffected mice and in −/− treated mice upon ICV and intraparenchymal injection. Scale bars, 100 μm. The blue dotted lines highlight the difference in CD68 immunoreactivity close to the ventricle (yellow box) versus farther away from the ventricle (dark blue box) in ICV-injected brains. Inset images were taken at higher magnification to highlight the differences in morphology of CD68-stained microglia. Scale bars, 20 μm. Post-acquisition processing was applied to all images and included adjustments to brightness and contrast and RGB curves using Adobe Photoshop CS6 to improve visibility and consistency in color tone. The histograms on the right show the area of CD68 immunoreactivity for each group, measured. *$p < 0.05$, **$p < 0.01$, ***$p < 0.001$; two-tailed, unpaired parametric t test. Values are shown as mean ± SEM (n = 3 mice per group). Mean with SEM. For p values, see Tables 1 and 2.

Table 1. p Values for the Quantitative Analysis of Mice that Received Postnatal ICV Injections of *N*-iNSCs and Controls

	Area	Comparison	p Value
		−/− vehicle versus unaffected	0.0040
	motor cortex	−/− treated (ICV) versus unaffected	0.0026
		−/− treated (ICV) versus −/− vehicle	0.3121
		−/− vehicle versus unaffected	0.0002
Figure 4	striatum	−/− treated (ICV) versus unaffected	0.0010
		−/− treated (ICV) versus −/− vehicle	0.0004
		−/− vehicle versus unaffected	0.0001
	ventral forebrain	−/− treated (ICV) versus unaffected	0.3397
		−/− treated (ICV) versus −/− vehicle	0.0001
		−/− vehicle versus unaffected	0.0020
	motor cortex	−/− treated (ICV) versus unaffected	0.0217
		−/− treated (ICV) versus −/− vehicle	0.8758
		−/− vehicle versus unaffected	0.0009
Figure S2	striatum	−/− treated (ICV) versus unaffected	0.0674
		−/− treated (ICV) versus −/− vehicle	0.4920
		−/− vehicle versus unaffected	0.0073
	ventral forebrain	−/− treated (ICV) versus unaffected	0.0209
		−/− treated (ICV) versus −/− vehicle	0.5806
		−/− vehicle versus unaffected	0.2262
	motor cortex	−/− treated (ICV) versus unaffected	0.1822
		−/− treated (ICV) versus −/− vehicle	0.5669
		−/− vehicle versus unaffected	0.0836
Figure S3	striatum	−/− treated (ICV) versus unaffected	0.0591
		−/− treated (ICV) versus −/− vehicle	0.5910
		−/− vehicle versus unaffected	0.4431
	ventral forebrain	−/− treated (ICV) versus unaffected	0.3597
		−/− treated (ICV) versus −/− vehicle	0.8442

Definition of statistical significance: $p < 0.05$.

compared with the vehicle-injected *Naglu*$^{-/-}$ mice regardless of the region analyzed, and thresholding image analysis confirmed this (Figure S2C, 1–3). These findings indicate that the *N*-iNSC cells had no effect on astrocyte activation in any brain region when injected via the ICV route, even in areas close to where the cells engrafted. In intraparenchymally grafted mice, there was no effect of the *N*-iNSC grafts on the level of GFAP immunoreactivity in the motor cortex, but, closer to the sites of engraftment, there was a significant decrease in GFAP staining in the striatum (1.7-fold reduction, p = 0.0155) and ventral forebrain (1.5-fold reduction, p = 0.0095) (Figure S2C, 1–3). Furthermore, the morphology of GFAP-positive astrocytes, having thinner, more palely stained processes and a smaller cell soma in intraparenchymally grafted *Naglu*$^{-/-}$ mice, resembled less activated astrocytes compared with the intensely stained GFAP-positive astrocytes with thickened processes and a hypertrophied cell body seen in other brain regions of these mice and in vehicle-injected *Naglu*$^{-/-}$ mice (Figure S2C).

Correction of Lysosomal Dysfunction and Storage Material

To evaluate the direct effect of the engrafted *N*-iNSCs on their lysosomal disease, we immunostained sections through the motor cortex, striatum, and ventral forebrain for the lysosomal membrane protein Lamp 1, which serves as a surrogate marker for lysosomal storage accumulation (Figures S3A and S3B).[32] As expected, Lamp 1 immunoreactivity was greater in vehicle-injected *Naglu*$^{-/-}$ mutant mice compared with unaffected heterozygous controls across all three areas assessed, but this did not reach significance because of the high variation within the vehicle-injected controls, with the exception of the striatum in the intraparenchymally grafted group (p = 0.0167, Figures S3B and S3C). In the ICV-grafted brains, there was no decrease in Lamp 1 immunoreactivity compared with vehicle-injected *Naglu*$^{-/-}$ mice in any of the brain regions assessed (Figure S3C, 1–3). In the intraparenchymally grafted brains, Lamp 1 staining was markedly decreased in the striatum and ventral forebrain and was not significantly different from unaffected animals (Figure S3C).

Taken together, our results suggest that intraparenchymal delivery of *N*-iNSCs results in a greater level of engraftment than the ICV route. These engrafted *N*-iNSCs are capable of secreting sufficient enzymes to correct disease-associated pathology, but this is limited to the area where they reside and has a greater effect on microglial activation than astrocytosis.

Long-Term Engraftment and Effect of *N*-iNSC on Neuropathology and Lysosomal Dysfunction

Based on our data from a 2-month survival time, a second cohort of mice was injected bilaterally into the parenchyma of the striatum at birth and sacrificed 9 months after transplantation to qualitatively assess the level and effects of longer-term engraftment. Ten mice per group were included in this analysis, and GFP-positive cells were detected in all but one animal. In the majority of the mice (seven of ten), GFP-positive cells could be detected partway along the rostrocaudal axis, with cells detected in the ventral and/or ventrolateral forebrain, along the fiber tracts of the *corpus callosum*, and the *substantia nigra*, as shown in Figure 5A, which shows a mouse with a high level of engraftment. In some brains, cells were also concentrated in other regions. In one animal, for example, a striking number of GFP-positive cells could be detected in the olfactory bulb and throughout the dorsorostral striatum, directly beneath the anterior forceps of the *corpus callosum* (data not shown).

Enzyme activity for the corresponding right hemisphere was evaluated for the six rostrocaudal 2-mm slices, as shown in Figure 5B. In our enzymatic assay, we combined sections 1 and 2 and sections 5 and 6. As expected, no NAGLU activity was detected in vehicle-injected *Naglu*$^{-/-}$ mice (Figure 5C). In the pool of sections 1 and 2, the averaged NAGLU activity in engrafted *Naglu*$^{-/-}$ mice reached a level almost 5-fold above the level of unaffected mice (Figure 5C). In sections 3 and 4 and the pool of 5 and 6, NAGLU activity in engrafted *Naglu*$^{-/-}$ mice was significantly greater than in vehicle-injected *Naglu*$^{-/-}$ mice ($p < 0.0001$ for sections 3, $p < 0.0001$ for section 4, and p = 0.0017 for sections 5 and 6) and reached approximately

Table 2. p Values for the Quantitative Analysis of Mice that Received Postnatal PAR Injections of *N*-iNSCs and Controls

Figure	Area	Comparison	p Value
Figure 4	motor cortex	−/− vehicle versus unaffected	0.0050
		−/− treated (PAR) versus unaffected	0.0099
		−/− treated (PAR) versus −/− vehicle	0.0307
	striatum	−/− vehicle versus unaffected	0.0120
		−/− treated (PAR) versus unaffected	0.0002
		−/− treated (PAR) versus −/− vehicle	0.0808
	ventral forebrain	−/− vehicle versus unaffected	0.0223
		−/− treated (PAR) versus unaffected	0.0006
		−/− treated (PAR) versus −/− vehicle	0.2432
Figure S2	motor cortex	−/− vehicle versus unaffected	0.0014
		−/− treated (PAR) versus unaffected	< 0.0001
		−/− treated (PAR) versus −/− vehicle	0.5702
	striatum	−/− vehicle versus unaffected	0.0023
		−/− treated (PAR) versus unaffected	0.0031
		−/− treated (PAR) versus −/− vehicle	0.0155
	ventral forebrain	−/− vehicle versus unaffected	0.0008
		−/− treated (PAR) versus unaffected	0.0445
		−/− treated (PAR) versus −/− vehicle	0.0095
Figure S3	motor cortex	−/− vehicle versus unaffected	0.0551
		−/− treated (PAR) versus unaffected	0.0296
		−/− treated (PAR) versus −/− vehicle	0.5046
	striatum	−/− vehicle versus unaffected	0.0167
		−/− treated (PAR) versus unaffected	0.1658
		−/− treated (PAR) versus −/− vehicle	0.1800
	ventral forebrain	−/− vehicle versus unaffected	0.1444
		−/− treated (PAR) versus unaffected	0.7773
		−/− treated (PAR) versus −/− vehicle	0.2273

Definition of statistical significance: $p < 0.05$.

10% of unaffected mice (Figure 5C). The lysosomal hexosaminidase enzyme is upregulated as a result of lysosomal dysfunction.[32,33] As expected, hexosaminidase was elevated in vehicle-injected *Naglu*$^{-/-}$ mice compared with unaffected mice in all sections (Figure 5D). Following engraftment in *Naglu*$^{-/-}$ mice, a partial reduction of hexosaminidase was achieved in the pool of sections 1 and 2, with a significant 1.2-fold reduction ($p = 0.0127$), in section 3, with a reduction of 1.2-fold ($p = 0.0166$), and a 1.3-fold reduction in section 4 ($p < 0.0001$), but no difference was observed for sections 5 and 6, which are farther away from the site of graft placement (Figure 5D).

To assess the effect of the *N*-iNCSs on MPS IIIB-associated neuropathology, we carried out qualitative analysis of microglial activation, astrocytosis, and lysosomal storage accumulation on representative sections from rostral (at the level of the isocortex and olfactory areas), middle (at the level where the fimbria of the hippocampus appears), and caudal (at the level of the midbrain) positions along the rostrocaudal axis of mice from each treatment group (Figure 6). For each section, images were taken at high magnification within three different regions. For the rostral section, images were taken in the dorsal isocortex, the ventral isocortex, and the olfactory area. For the middle section, images were taken in the motor cortex, striatum, and ventral forebrain. In the caudal section, the dorsal cortex, dorsal midbrain, and ventral midbrain were imaged. In general, all neuropathological hallmarks (CD68, GFAP, and Lamp 1) exhibited by *Naglu*$^{-/-}$ mice were more pronounced at 9 months of age compared with 2 months (Figures 6A–6C, low magnification). However, CD68 (Figure 6A) and GFAP (Figure 6B) immunoreactivity in intraparenchymally grafted *Naglu*$^{-/-}$ mice were markedly reduced at all three rostrocaudal levels examined and were largely indistinguishable from the appearance of these markers at the corresponding level of the brain in unaffected mice (Figure 6). However, in the middle and caudal sections (farther away from graft placement), some activated microglia and astrocytes could still be detected in the grafted *Naglu*$^{-/-}$ mice (Figures 6A and 6B, Middle and Caudal, box 1).

Lamp 1 immunoreactivity was markedly increased in untreated 9-month-old *Naglu*$^{-/-}$ mice compared with unaffected heterozygous controls throughout all three levels along the rostrocaudal axis (Figure 6C). At 2 months of age, Lamp 1 staining in vehicle-injected *Naglu*$^{-/-}$ mice was at a relatively low level and highly variable, making it difficult to see significant changes between vehicle-injected and intraparenchymally grafted *Naglu*$^{-/-}$ mice (Figure S3C, 1–3). However, 9 months post-transplantation there was a consistent and more generalized decrease in Lamp 1 immunoreactivity in all three rostrocaudal sections (Figure 6C). As seen for CD68 and GFAP, the level of Lamp 1 immunoreactivity in the motor cortex and dorsal cortex farther away from the graft placement in the middle and caudal sections, respectively, remained similar to vehicle-treated *Naglu*$^{-/-}$ mice (Figure 6C).

Taken together, these data provide evidence that grafted *N*-iNSCs can persist for prolonged periods of time and not only still secrete significantly elevated levels of NAGLU enzyme but also have pronounced effects on the neuropathology associated with MPS IIIB in mice.

DISCUSSION

MPS IIIB is caused by a deficiency in NAGLU activity, resulting in accumulation of heparan sulfate glycosaminoglycan. Although the exact mechanism of disease is unknown, this enzyme deficiency leads to lysosomal pathology and a pronounced neuroimmune response. Unlike recombinant NAGLU,[20,21,32] we showed that NAGLU produced by our *N*-iNSCs *in vitro* can be efficiently endocytosed by deficient cells via M6PRs (Figure 2) and are capable of correcting microglial activation, astrocytosis, and lysosomal storage accumulation *in vivo*.

Two injection methods were used in the 2 months after grafting experiments to determine the best route of delivery according to the level of invasiveness and how much engraftment is

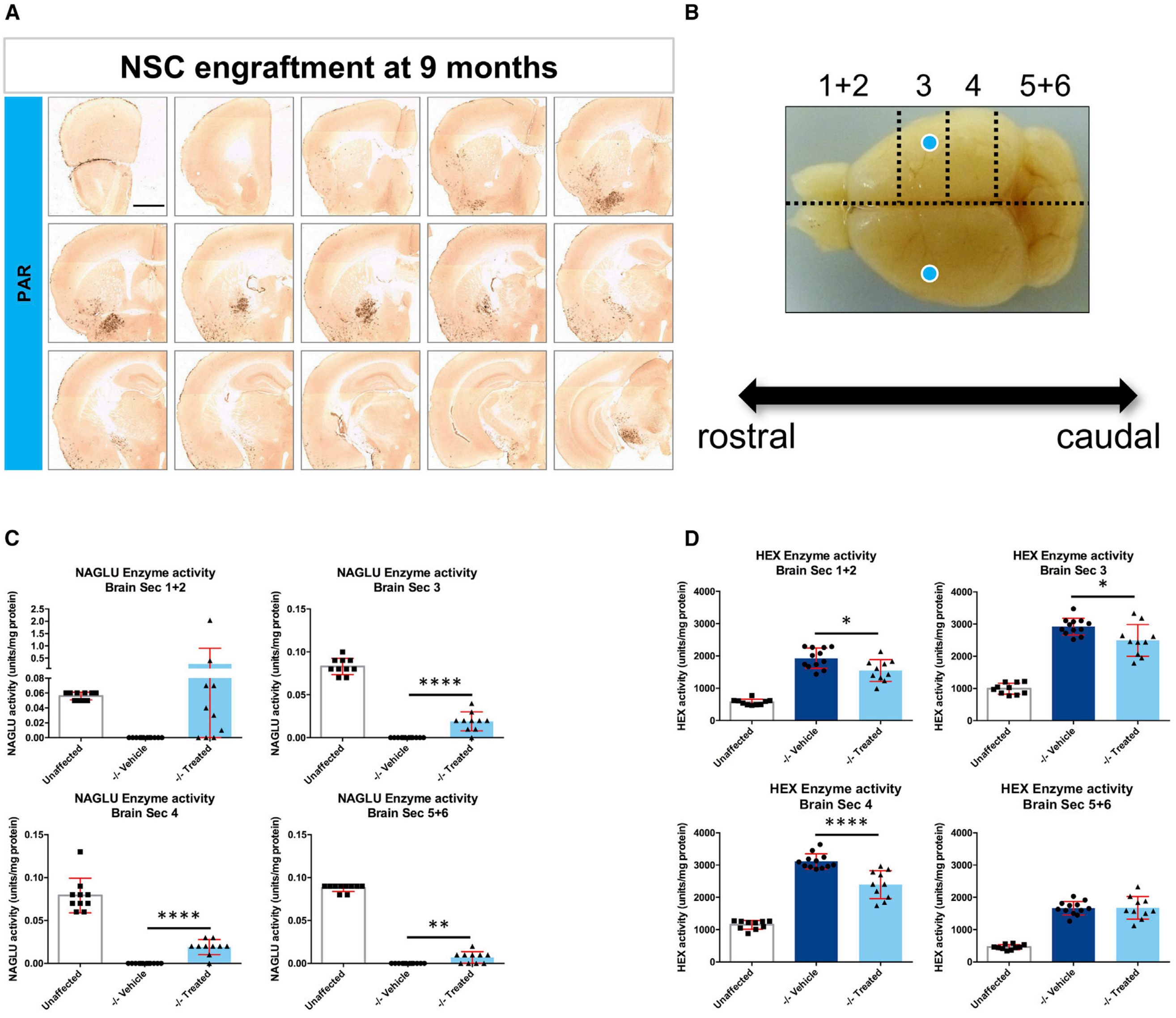

Figure 5. NAGLU Corrected N-iNSCs Grafting and Characterization of Enzyme Activity at 9 Months

(A) Immunohistological staining of 40-μm representative sections for GFP, shown at low magnification, following PAR injection of *N*-iNSCs into *Naglu*$^{-/-}$ mice. Post-acquisition processing included adjustments to brightness and contrast and RGB curves using Adobe Photoshop CS6 to improve visibility and consistency in color tone. Scale bar, 1 mm. (B) Schematic diagram depicting how the brains were divided. Brains were dissected sagittally along the midline first. One brain hemisphere was for histology, and the other brain hemisphere was further sectioned into 2-mm-thick coronal slices in each animal. For NAGLU and hexosaminidase (HEX) activity assays, brain sections 1 and 2 and 5 and 6 were pooled. The blue dots represent the approximate intraparenchymal injection sites. (C and D) Enzymatic activity of NAGLU (C) and HEX (D), respectively, in *Naglu*$^{-/-}$ mice with *N*-iNSCs (−/− treated) or saline (−/− vehicle) compared with heterozygous control mice (unaffected). All bars show mean ± SD (n = 10–12 mice per group). Mean with SD. Two-tailed, unpaired parametric t tests were used between (−/− treated) or saline (−/− vehicle) groups.*p < 0.05, **p < 0.01, ****p < 0.0001.

achieved: injection into the ventricles (ICV) or directly into the striatum (intraparenchymal). It has been shown previously that some NSCs injected into the ventricles (ICV) are capable of widespread engraftment and can differentiate into neurons or glia.[16] In comparison, the level of engraftment achieved 2 months after grafting using ICV injection in our experiment was low (Figure 3). A greater level of engraftment was achieved via the intraparenchymal route at this time point, but the majority of these cells were detected within a concentrated region of the ventral forebrain (Figure 3). Furthermore, the degree of iNSC engraftment was inconsistent in this cohort of mice. Not only was the level of engraftment variable, as assessed by GFP immunostaining, but so was the level of NAGLU activity produced by these grafts. Some animals possessed activity greater than or equal to heterozygous controls, whereas others showed little to no NAGLU activity. We speculate that the specific location of the cells accounts for this variation.

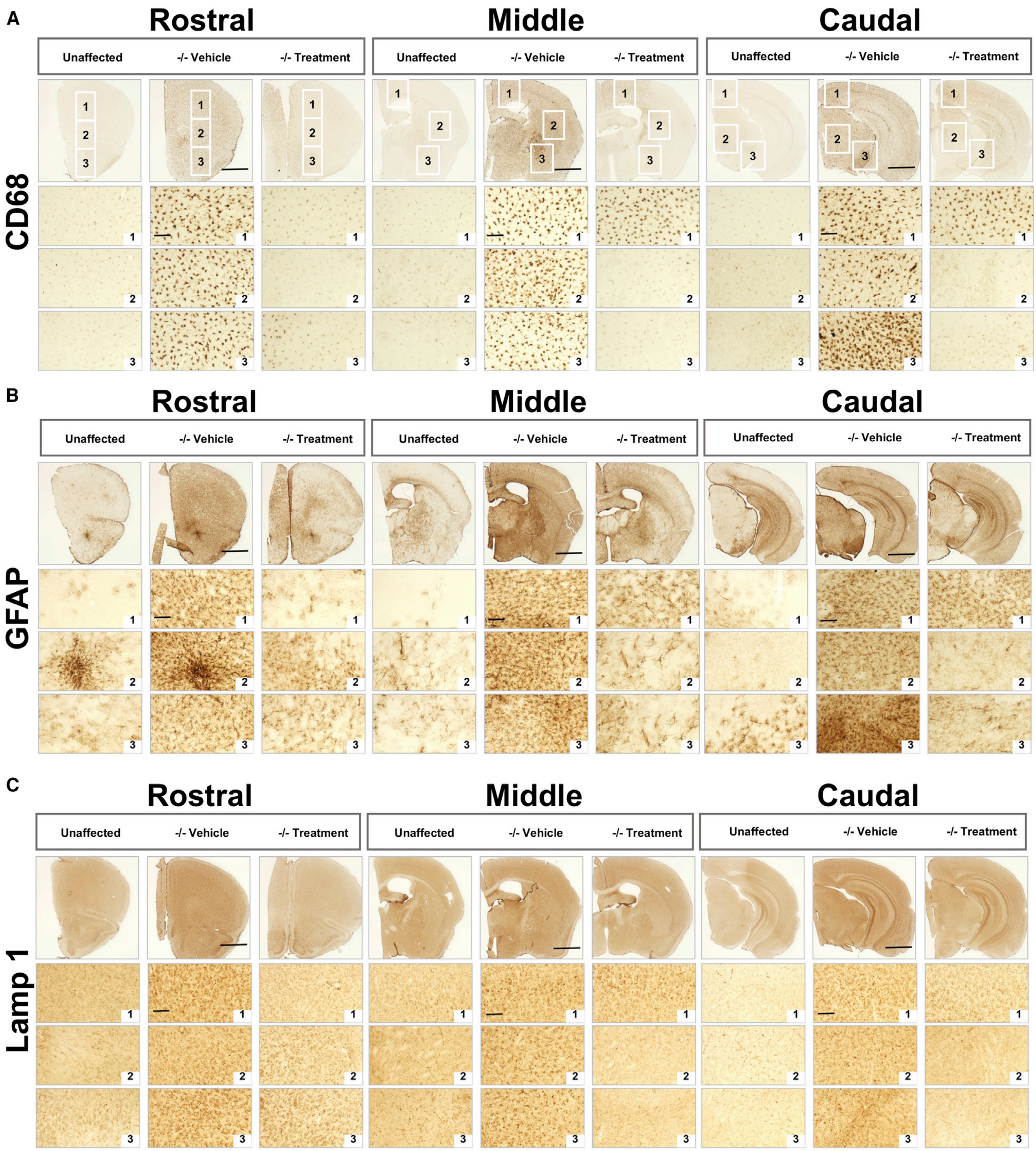

Figure 6. Correction of Neuropathology and Storage Accumulation in Naglu$^{-/-}$ Mice Treated with Neural Stem Cells Overexpressing NAGLU *N*-iNSCs at 9 Months

(A–C) Representative bright-field images of half-brain coronal sections from heterozygous control mice (unaffected) and mice injected with saline (−/− vehicle) or *N*-iNSCs (−/− treated). Images were taken at three levels along the rostrocaudal axis (rostral, middle, and caudal) and immunohistochemically stained for CD68 (A), GFAP (B), and Lamp 1 (C), respectively. Numbered boxes show images taken at higher magnification from three specific areas within each section (boxes 1–3, outlined at lower magnification, top). The same three areas were analyzed for GFAP and Lamp 1. Post-acquisition processing was applied to all images and included adjustments to brightness and contrast and RGB curves using Adobe Photoshop CS6 to improve visibility and consistency in color tone. Scale bars, 1 mm.

The level of engraftment and distribution of cells was different in our long-term study than 2 months after grafting, with more cells detected and a broader dispersion throughout the brain being achieved at 9 months. It seems likely that this discrepancy is due to a better initial engraftment of *N*-iNSCs in the 9-month survival experiment. However, altered proliferation and or differentiation and spread of these *N*-iNSCs cannot be excluded in a more diseased MPS IIIB brain, and further experiments will be needed to distinguish between these possibilities.

Based on the 9 months grafting experiments, our data show that the ability of engrafted cells to correct pathology depends primarily on the total level of engraftment rather than the precise distribution of the cells because we observed almost complete correction in some regions with low engraftment. Two months post-grafting, we documented the ability of iNSCs to correct nearby areas of pathology and lysosomal storage accumulation when engraftment is localized. Interestingly, as reported previously,[34] neuroinflammation (microglial activation and level of cytokines) is established even before lysosomal storage accumulation reaches a significant level. Our data suggest that the underlying disease mechanisms that lead to the neuroimmune response seen in MPS IIIB mice may not be a direct result of storage accumulation and that overall neuropathology results from a number of different events that converge at a particular pathological endpoint. It is also plausible that a deficiency in NAGLU has other consequences, aside from failing to simply break down heparan sulfate. In many neurodegenerative diseases, local activation of resident microglia and astrocytes and infiltration of peripheral leucocytes as part of a normally protective immune reaction can be toxic to neurons.[35,36] In other diseases, such as Cln3 disease, glia are themselves affected by the disease and can harm neurons directly.[37]

Upregulation of GFAP and activation of astrocytes in the MPS IIIB mouse brain has been widely reported.[31,38] However, at 2 months, in comparison with microglial activation, the ability of the engrafted cells to temper astrocyte activation was less evident in the area where they had immediately engrafted and even less so at a distance (Figure S2). This result could have several interpretations. For example, it is possible that microglia require a lower threshold of NAGLU to attenuate their activation or that microglia are more sensitive to anti-inflammatory signals released by the injected NSCs. Although we have not evaluated the anti-inflammatory properties of grafted *N*-NSCs in this study, it has been reported that NSCs can exert this function.[39]

As expected, our data showed that, at 9 months of age, *Naglu*$^{-/-}$ mice display a more pronounced storage burden, microglial activation, and astrocytosis than at a younger age. Transplanted cells were able to provide at least 10% of heterozygote-level NAGLU activity throughout the brain, consequently almost completely correcting lysosomal storage accumulation and neuropathology. However, even 21% of heterozygote-level NAGLU activity was not adequate for complete dorsoventral correction of pathology, with the dorsal region of the cortex showing the least correction. This result could be explained by the *corpus callosum* potentially acting as a physical barrier for the diffusion of NAGLU enzyme to reach the cortex or the considerable distance of the grafted cells from this cortical region. However, in at least one animal, we did unexpectedly observe complete correction of the olfactory bulb, which is farthest away from the majority of engrafted cells. This suggests that the relationship between the level and sites of engraftment and the ability of *N*-iNSCs to correct pathology is a complex one that is not fully understood. Nevertheless, our data suggest that the relative amount and spread of grafted cells, rather than their particular location, has the greatest influence on disease-related pathology. Therefore, we speculate that any strategy that promotes more widespread engraftment of *N*-iNSCs would improve their efficacy.

Our data provide evidence toward the potential of using NSCs overexpressing NAGLU as a means to treat MPS IIIB. Indeed, NSC therapies are in clinical trials to treat Parkinson's disease, stroke, cancer, amyotrophic lateral sclerosis (ALS), spinal cord injury, and Pelizaeus-Merzbacher disease.[40] Optimization of our injection procedure and the properties of the NSC lines used to lead to greater engraftment will be needed if this goal is to be realized.

MATERIALS AND METHODS

Lentivirus Production

The human *NAGLU* gene was engineered from a previously published *NAGLU-IGF2*[20] sequence and further cloned into a lentiviral backbone plasmid, pSAM-GFP (a gift from Dr. Michael Kyba, University of Minnesota) at the XhoI and NotI sites. The resulting expression vector was named pSAM-NAGLU-GFP. The lentiviral supernatant was produced with 293T cells (American Type Culture Collection, Manassas, Virginia) as described previously.[41]

Cellular Reprogramming

Naglu$^{-/-}$ MEFs were transduced with the reprogramming factors pMXs-*OCT3/4*, pMXs-*SOX2*, pMXs-*KLF4*, and pMXs-c-*MYC* (Addgene, Cambridge, MA) as reported previously.[18] MEFs were cultured until individual iPSC colonies were formed. Colonies were picked and grown individually on a feeder layer of MEFs treated with mitomycin C (Roche, Indianapolis, IN) as described before.[41] Characterization was performed using alkaline phosphatase staining (Cell Biolabs, San Diego, CA) and immunofluorescence for the presence of Oct4 (see Immunocytochemistry).

Directed Differentiation of iPSCs to iNSCs

iPSCs were grown feeder-free at a density of 8.0×10^4 cells/well in embryonic stem cell (ESC) medium (knockout DMEM with 10% fetal bovine serum [FBS] and 500 U/mL leukemia inhibitory factor; Millipore, Burlington, MA). The next day, the medium was changed to NSC medium (DMEM/F12 supplemented with B27 without vitamin A and N2; Gibco, Carlsbad, CA) and grown for 12 days. On day 12, NSC medium, 20 ng/mL of epidermal growth factor (EGF), and 20 ng/mL basic fibroblast growth factor (bFGF) were added (PeproTech, Rocky Hill, NJ). Cells were expanded in NSC medium + EGF + bFGF as monolayers.[19]

Real-Time qPCR

Total RNA was extracted from iPSC clones with Trizol (Thermo Fisher Scientific, Waltham, MA), and cDNA was generated using 1 μg DNase-treated RNA with oligo-dT primers and ThermoScript reverse transcriptase (Thermo Fisher Scientific, Waltham, MA) according to the manufacturer's instructions. PCR reactions were run in triplicate and quantified on a PikoReal real-time PCR system (Thermo Fisher Scientific, Waltham, MA) using TaqMan real-time PCR premixtures (Bioline, Taunton, MA) and the pre-made TaqMan probes Nestin (Mm00450205_m1) and GAPDH (Mm99999915-g1) (Applied Biosystems, Foster City, CA). Results were expressed as gene expression level compared with GAPDH.

Enzyme Activity Assays

The catalytic activity of NAGLU was determined by hydrolysis of the fluorogenic substrate 4-methylumbelliferyl-*N*-acetyl-α-glucosaminide (EMD Millipore, Burlington, MA) with a final concentration of 0.1 mM substrate in the incubation mixture as described previously.[20] The catalytic activity of β-hexosaminidase (combined A and B isoforms) was determined by hydrolysis of 4-methylumbelliferyl-*N*-acetyl-β-glucosaminide (EMD Millipore, Burlington, MA) using 1.25 mM substrate in the incubation mixture. A unit of activity is defined as the release of 1 nmol of 4-methylumbelliferone (4MU) per hour at 37°C. Protein concentration was estimated by the Bradford method, using BSA (Bio-Rad, Hercules, CA) as the standard. Fluorescence was measured on a microplate reader (Spectramax Paradigm, Molecular Devices, San Jose, CA) with λ_{ex} 365 nm and λ_{em} 445 nm. Intracellular NAGLU activity is presented as units per milligram of protein, and secreted NAGLU is presented as units per milliliter.

Endocytosis of Enzyme in Cell Culture

Naglu$^{-/-}$ iNSCs were plated onto 12-well tissue culture plates at 1×10^5 cells per well and subsequently incubated with filtered (0.45 μm) supernatants that were taken from T25 flasks containing 1×10^6 *N*-i*Naglu*$^{-/-}$ NSCs. After 4 hr, the cells were washed and lysed with mammalian protein extraction reagent (M-PER, Thermo Fisher Scientific, Waltham, MA), and the lysates and filtered supernatant were assayed for enzyme activity as described above. Supernatants and cell lysates from *Naglu*$^{-/-}$ iNSCs served as negative controls, and supernatants and cell lysates from WT iPSCs served as positive controls.

LysoTracker Staining

LysoTracker (Thermo Fisher Scientific, Waltham, MA) staining was performed following the manufacturer's procedure. Briefly, cells were stained with 150 nM LysoTracker in relevant culture medium for 1 hr at 37°C and 5% CO_2. Following incubation, cells were washed twice with PBS (137 mM NaCl, 2.7 mM KCl, 10 mM Na_2HPO_4, and 1.76 mM KH_2PO_4 [pH 7.4]) and analyzed by FACS.

Immunocytochemistry

Cells were plated on glass coverslips and grown in appropriate media. Cells were fixed with 4% paraformaldehyde. Primary antibodies used were monoclonal mouse anti-Olig2 (Thermo Fisher Scientific, Waltham, MA; catalog no. MA5-15810), monoclonal mouse anti-Nestin (Invitrogen Carlsbad, CA; catalog no. 14584380), polyclonal rabbit anti-MAP2 (Thermo Fisher Scientific, Waltham, MA; catalog no. PA5-17646), polyclonal rabbit anti-GFAP (Agilent Technologies, Santa Clara, CA; catalog no. Z0334), and mouse anti-Tuj1 (The Developmental Studies Hybridoma Bank [DSHB], Iowa City, IA; catalog no. 6g7) (all 1:400). The following secondary antibodies were selected according to the primary antibody used: goat anti-mouse Alexa 488, catalog no. A10640 (Olig2); goat anti-mouse Alexa 555, catalog no. A21424 (Nestin); goat anti-rabbit Alexa 4888, catalog no. A11008 (MAP2); goat anti-rabbit Alexa 555, catalog no. A21428 (GFAP); and goat anti-mouse Alexa 555, catalog no. A21424 (Tuj-1) (all from Molecular Probes, Eugene, OR; 1:1,000 dilution).

Experimental Animals

Animal experiments were approved by the Institutional Animal Care and Use Committee at the Los Angeles Biomedical Research Institute at Harbor-UCLA Medical Center, which is accredited by the Association for Assessment and Accreditation of Laboratory Animal Care (AAALAC). The *Naglu*$^{-/-}$ knockout mouse was a gift from Dr. Neufeld from the University of California, Los Angeles (UCLA) and was back-bred onto C57BL6/J.[42] Genotyping was performed with the following primers: NAG5′, 5′-TGGACCTGTTTGCTGAAAGC-3′; NAG3′, 5′-CAGGCCATCAAATCTGGTAC-3′; Neo5′, 5′-TGGGAT CGGCCATTGAACAA-3′; and Neo3′, 5′-CCTTGAGCCTGGCGA ACAGT-3′. *Naglu*$^{+/-}$ females were crossed with *Naglu*$^{-/-}$ males to obtain homozygous affected mice and heterozygous controls. Experiments were performed on age-matched mice (usually littermates) of either gender.

Injection of NSCs

2.5×10^5 NAGLU-overexpressing NSCs (*N*-iNSC) in 3 μl of PBS or PBS alone were injected bilaterally on post-natal day 0 (P0) or P1 neonates of *Naglu*$^{-/-}$ mice under cryo-anesthesia by Hamilton syringe with 32G needles. The injection site for ICV injection was about 2.5 mm between the bregma and the eyes and 2 mm away laterally from the sagittal suture and 2.5-mm depth. For intraparenchymal injection, it was about 3.0 mm between the bregma and the eyes and 4.0 mm away laterally from the sagittal suture and 3.0 mm depth.

Percoll Gradient Centrifugation

To isolate lysosomes, a Percoll gradient was constructed as follows: one part 10× PBS was added to nine parts Percoll (Sigma-Aldrich, St. Louis, MO) to make a stock isotonic solution. Then the stock isotonic Percoll was diluted to a 30% concentration in PBS, and Hibernate A (GIBCO, Carlsbad, CA) was placed above the 30% Percoll layer.

Brains were digested using a 1:1 mixture of Accutase (STEMCELL Technologies, Vancouver, Canada) and trypsin (GIBCO, Carlsbad, CA) for 1 hr at 37°C. Mechanical dissociation was achieved using 18G needles followed by 25G needles for 10 repetitions each. Brain

homogenates were added to the Percoll gradient and centrifuged at 1,200 × g for 45 min. Debris in the top layer was aspirated, the Percoll gradient was diluted in PBS, and samples were centrifuged at $3,000 \times g$. Cells were then processed for FACS or lysed for enzymatic assays.

Tissue Harvesting, Processing, and Histological Staining

After 2 and 9 months post-transplantation, brains were dissected sagittally along the midline, and left hemispheres were post-fixed overnight at 4°C in 4% paraformaldehyde (PFA) before cryoprotection at 4°C in a solution of 30% sucrose in Tris-buffered saline. The right brain hemispheres were further sectioned into 2 mm-thick coronal slices using an adult mouse brain slicer matrix (Zivic Instruments, Pittsburgh, PA) and rapidly frozen and stored at −80°C until performing NAGLU and β-hexosaminidase activity assays. 40-μm frozen coronal sections were cut through the rostrocaudal extent of the cortical mantle (Microm HM 430 freezing microtome, Thermo Fisher Scientific, Waltham, MA). Sections were collected in a cryoprotectant solution (tris-buffered saline [TBS]/30% ethylene glycol/15% sucrose/0.05% sodium azide) and stored at 4°C before histological processing.[43–45]

Immunohistochemistry for Assessing Stem Cell Engraftment, Glial Markers, and Storage Accumulation

To examine the level of stem cell engraftment, the extent of glial activation and storage material accumulation, adjacent one-in-six series of free-floating frozen sections were immunohistochemically stained using the following standard immunoperoxidase protocol, as described previously.[43–45] Anti-GFP (1:10,000 dilution, Invitrogen, catalog no. A10259), polyclonal rabbit anti-GFAP (1:8,000 dilution, Agilent Technologies, Santa Clara, CA; catalog no. Z0334), rat anti-CD68 (1:2,000, Bio-Rad, Hercules, CA; catalog no. MCA1957), and rat anti-Lamp 1 (1:4,000, DSHB, Iowa City, IA; catalog no. 1D4B), and rabbit immunoglobulin G (IgG) fraction were used, followed by the appropriate secondary antiserum: biotinylated goat anti-rabbit IgG (1:1,000, Vector Laboratories, Burlingame, CA; catalog no. BA-1000), biotinylated swine anti-rabbit IgG (GFAP, Agilent Technologies, Santa Clara, CA; catalog no. E0353), biotinylated rabbit anti-rat IgG (CD68, Vector Laboratories, Burlingame, CA; catalog no. BA-4001), and biotinylated goat anti-rat IgG (Lamp 1, Vector Laboratories, Burlingame, CA; catalog no. BA-9400), all 1:1,000 dilution. Sections were incubated in an avidin-biotin-peroxidase complex kit (Vectastain Elite ABC Kit, Vector Laboratories, Burlingame, CA). Immunoreactivity was visualized by incubation in 0.05% 3', 3'-diaminobenzidine (DAB; Sigma-Aldrich, St. Louis, MO) and 0.001% H_2O_2 in TBS for 10 min, a time that represents saturation for this reaction.

Quantitative Analysis of Glial Phenotype and Storage Accumulation

To analyze the degree of glial activation and storage material accumulation in the motor cortex, striatum, and ventral forebrain of sections stained for GFAP, CD68, and Lamp 1, an adapted semi-automated thresholding image analysis method[43,46] was used with Image-Pro Premier software (Media Cybernetics, Rockville, MD). Each region of interest was defined according to neuroanatomical landmarks described by Paxinos and Franklin[47] across three sections. The sections selected included the section corresponding to the greatest level of engraftment and the sections immediately before and after.

Statistical Analysis

All statistical analyses were performed using Prism software (GraphPad, La Jolla, CA). A two-tailed, unpaired, parametric t test was used when two groups were compared. Results were considered statistically significant when $p < 0.05$. Unless otherwise stated, statistical comparisons are between untreated MPS IIIB mice and unaffected heterozygous controls and the treatment groups and untreated MPS IIIB mice. One-way ANOVA was used to compare groups greater than two.

SUPPLEMENTAL INFORMATION

Supplemental Information includes three figures and can be found with this article online at https://doi.org/10.1016/j.omtm.2018.06.005.

AUTHOR CONTRIBUTIONS

Conceptualization, M.I. and P.I.D.; Methodology, M.I., P.I.D., and J.D.C.; Validation, Y.P., D.C., and S.-h.K.; Formal Analysis, Y.P., D.C., and S.-h.K.; Investigation, Y.P., D.C., S.-h.K., S.Q.L., and V.S.; Resources, M.I., P.I.D., and J.D.C.; Data Curation, Y.P., D.C, and S.-h.K.; Writing – Original Draft, Y.P. and D.C.; Writing – Review & Editing, Y.P., M.I., P.I.D., J.D.C., and S.-h.K.; Visualization, Y.P., D.C., and S.-h.K.; Supervision, M.I., P.I.D., and J.D.C.; Project Administration, M.I. and P.I.D.; Funding Acquisition, P.I.D. and M.I.

ACKNOWLEDGMENTS

We thank the members of Dr. Cooper's Pediatric Storage Disorders Laboratory for discussions and technical support. We thank Dr. Neufeld for providing Naglu knockout mice and Dr. Kyba for providing the lentivector pSam. We also thank Sean Ekins from Phoenix Nest Inc. for participating in discussions. This work was supported by grants from the NINDS (1R41NS092221-0181, 1R01 NS088766, and R21NS096044) and a traineeship from 5T32 GM8243-28 (to S.-h.K.).

REFERENCES

1. Valstar, M.J., Ruijter, G.J., van Diggelen, O.P., Poorthuis, B.J., and Wijburg, F.A. (2008). Sanfilippo syndrome: a mini-review. J. Inherit. Metab. Dis. *31*, 240–252.
2. Sarrazin, S., Lamanna, W.C., and Esko, J.D. (2011). Heparan sulfate proteoglycans. Cold Spring Harb. Perspect. Biol. *3*, a004952.
3. Andrade, F., Aldámiz-Echevarría, L., Llarena, M., and Couce, M.L. (2015). Sanfilippo syndrome: Overall review. Pediatr. Int. *57*, 331–338.
4. Kakkis, E.D., Muenzer, J., Tiller, G.E., Waber, L., Belmont, J., Passage, M., Izykowski, B., Phillips, J., Doroshow, R., Walot, I., et al. (2001). Enzyme-replacement therapy in mucopolysaccharidosis I. N. Engl. J. Med. *344*, 182–188.
5. Muenzer, J., Gucsavas-Calikoglu, M., McCandless, S.E., Schuetz, T.J., and Kimura, A. (2007). A phase I/II clinical trial of enzyme replacement therapy in mucopolysaccharidosis II (Hunter syndrome). Mol. Genet. Metab. *90*, 329–337.
6. Hendriksz, C.J., Burton, B., Fleming, T.R., Harmatz, P., Hughes, D., Jones, S.A., Lin, S.P., Mengel, E., Scarpa, M., Valayannopoulos, V., et al.; STRIVE Investigators (2014).

Efficacy and safety of enzyme replacement therapy with BMN 110 (elosulfase alfa) for Morquio A syndrome (mucopolysaccharidosis IVA): a phase 3 randomised placebo-controlled study. J. Inherit. Metab. Dis. *37*, 979–990.

7. Harmatz, P., Giugliani, R., Schwartz, I., Guffon, N., Teles, E.L., Miranda, M.C., Wraith, J.E., Beck, M., Arash, L., Scarpa, M., et al.; MPS VI Phase 3 Study Group (2006). Enzyme replacement therapy for mucopolysaccharidosis VI: a phase 3, randomized, double-blind, placebo-controlled, multinational study of recombinant human N-acetylgalactosamine 4-sulfatase (recombinant human arylsulfatase B or rhASB) and follow-on, open-label extension study. J. Pediatr. *148*, 533–539.

8. Barton, N.W., Brady, R.O., Dambrosia, J.M., Di Bisceglie, A.M., Doppelt, S.H., Hill, S.C., Mankin, H.J., Murray, G.J., Parker, R.I., Argoff, C.E., et al. (1991). Replacement therapy for inherited enzyme deficiency–macrophage-targeted glucocerebrosidase for Gaucher's disease. N. Engl. J. Med. *324*, 1464–1470.

9. Heldermon, C.D., Qin, E.Y., Ohlemiller, K.K., Herzog, E.D., Brown, J.R., Vogler, C., Hou, W., Orrock, J.L., Crawford, B.E., and Sands, M.S. (2013). Disease correction by combined neonatal intracranial AAV and systemic lentiviral gene therapy in Sanfilippo Syndrome type B mice. Gene Ther. *20*, 913–921.

10. Ribera, A., Haurigot, V., Garcia, M., Marcó, S., Motas, S., Villacampa, P., Maggioni, L., León, X., Molas, M., Sánchez, V., et al. (2015). Biochemical, histological and functional correction of mucopolysaccharidosis type IIIB by intra-cerebrospinal fluid gene therapy. Hum. Mol. Genet. *24*, 2078–2095.

11. Fu, H., Kang, L., Jennings, J.S., Moy, S.S., Perez, A., Dirosario, J., McCarty, D.M., and Muenzer, J. (2007). Significantly increased lifespan and improved behavioral performances by rAAV gene delivery in adult mucopolysaccharidosis IIIB mice. Gene Ther. *14*, 1065–1077.

12. Sivakumur, P., and Wraith, J.E. (1999). Bone marrow transplantation in mucopolysaccharidosis type IIIA: a comparison of an early treated patient with his untreated sibling. J. Inherit. Metab. Dis. *22*, 849–850.

13. Holley, R.J., Ellison, S.M., Fil, D., O'Leary, C., McDermott, J., Senthivel, N., Langford-Smith, A.W.W., Wilkinson, F.L., D'Souza, Z., Parker, H., et al. (2018). Macrophage enzyme and reduced inflammation drive brain correction of mucopolysaccharidosis IIIB by stem cell gene therapy. Brain *141*, 99–116.

14. Borger, D.K., McMahon, B., Roshan Lal, T., Serra-Vinardell, J., Aflaki, E., and Sidransky, E. (2017). Induced pluripotent stem cell models of lysosomal storage disorders. Dis. Model. Mech. *10*, 691–704.

15. Griffin, T.A., Anderson, H.C., and Wolfe, J.H. (2015). Ex vivo gene therapy using patient iPSC-derived NSCs reverses pathology in the brain of a homologous mouse model. Stem Cell Reports *4*, 835–846.

16. Snyder, E.Y., Taylor, R.M., and Wolfe, J.H. (1995). Neural progenitor cell engraftment corrects lysosomal storage throughout the MPS VII mouse brain. Nature *374*, 367–370.

17. Tamaki, S.J., Jacobs, Y., Dohse, M., Capela, A., Cooper, J.D., Reitsma, M., He, D., Tushinski, R., Belichenko, P.V., Salehi, A., et al. (2009). Neuroprotection of host cells by human central nervous system stem cells in a mouse model of infantile neuronal ceroid lipofuscinosis. Cell Stem Cell *5*, 310–319.

18. Takahashi, K., and Yamanaka, S. (2006). Induction of pluripotent stem cells from mouse embryonic and adult fibroblast cultures by defined factors. Cell *126*, 663–676.

19. Onorati, M., Camnasio, S., Binetti, M., Jung, C.B., Moretti, A., and Cattaneo, E. (2010). Neuropotent self-renewing neural stem (NS) cells derived from mouse induced pluripotent stem (iPS) cells. Mol. Cell. Neurosci. *43*, 287–295.

20. Kan, S.H., Troitskaya, L.A., Sinow, C.S., Haitz, K., Todd, A.K., Di Stefano, A., Le, S.Q., Dickson, P.I., and Tippin, B.L. (2014). Insulin-like growth factor II peptide fusion enables uptake and lysosomal delivery of α-N-acetylglucosaminidase to mucopolysaccharidosis type IIIB fibroblasts. Biochem. J. *458*, 281–289.

21. Zhao, K.W., and Neufeld, E.F. (2000). Purification and characterization of recombinant human alpha-N-acetylglucosaminidase secreted by Chinese hamster ovary cells. Protein Expr. Purif. *19*, 202–211.

22. Weber, B., Hopwood, J.J., and Yogalingam, G. (2001). Expression and characterization of human recombinant and alpha-N-acetylglucosaminidase. Protein Expr. Purif. *21*, 251–259.

23. Pol, A., Luetterforst, R., Lindsay, M., Heino, S., Ikonen, E., and Parton, R.G. (2001). A caveolin dominant negative mutant associates with lipid bodies and induces intracellular cholesterol imbalance. J. Cell Biol. *152*, 1057–1070.

24. Xu, M., Liu, K., Swaroop, M., Sun, W., Dehdashti, S.J., McKew, J.C., and Zheng, W. (2014). A phenotypic compound screening assay for lysosomal storage diseases. J. Biomol. Screen. *19*, 168–175.

25. Vázquez, P., Arroba, A.I., Cecconi, F., de la Rosa, E.J., Boya, P., and de Pablo, F. (2012). Atg5 and Ambra1 differentially modulate neurogenesis in neural stem cells. Autophagy *8*, 187–199.

26. Glick, D., Barth, S., and Macleod, K.F. (2010). Autophagy: cellular and molecular mechanisms. J. Pathol. *221*, 3–12.

27. Donsante, A., Levy, B., Vogler, C., and Sands, M.S. (2007). Clinical response to persistent, low-level beta-glucuronidase expression in the murine model of mucopolysaccharidosis type VII. J. Inherit. Metab. Dis. *30*, 227–238.

28. Garcia, A.R., DaCosta, J.M., Pan, J., Muenzer, J., and Lamsa, J.C. (2007). Preclinical dose ranging studies for enzyme replacement therapy with idursulfase in a knock-out mouse model of MPS II. Mol. Genet. Metab. *91*, 183–190.

29. Amor, S., Puentes, F., Baker, D., and van der Valk, P. (2010). Inflammation in neurodegenerative diseases. Immunology *129*, 154–169.

30. Langford-Smith, A., Wilkinson, F.L., Langford-Smith, K.J., Holley, R.J., Sergijenko, A., Howe, S.J., Bennett, W.R., Jones, S.A., Wraith, J., Merry, C.L., et al. (2012). Hematopoietic stem cell and gene therapy corrects primary neuropathology and behavior in mucopolysaccharidosis IIIA mice. Mol. Ther. *20*, 1610–1621.

31. Wilkinson, F.L., Holley, R.J., Langford-Smith, K.J., Badrinath, S., Liao, A., Langford-Smith, A., Cooper, J.D., Jones, S.A., Wraith, J.E., Wynn, R.F., et al. (2012). Neuropathology in mouse models of mucopolysaccharidosis type I, IIIA and IIIB. PLoS ONE *7*, e35787.

32. Kan, S.H., Aoyagi-Scharber, M., Le, S.Q., Vincelette, J., Ohmi, K., Bullens, S., Wendt, D.J., Christianson, T.M., Tiger, P.M., Brown, J.R., et al. (2014). Delivery of an enzyme-IGFII fusion protein to the mouse brain is therapeutic for mucopolysaccharidosis type IIIB. Proc. Natl. Acad. Sci. USA *111*, 14870–14875.

33. Aoyagi-Scharber, M., Crippen-Harmon, D., Lawrence, R., Vincelette, J., Yogalingam, G., Prill, H., Yip, B.K., Baridon, B., Vitelli, C., Lee, A., et al. (2017). Clearance of Heparan Sulfate and Attenuation of CNS Pathology by Intracerebroventricular BMN 250 in Sanfilippo Type B Mice. Mol. Ther. Methods Clin. Dev. *6*, 43–53.

34. Ausseil, J., Desmaris, N., Bigou, S., Attali, R., Corbineau, S., Vitry, S., Parent, M., Cheillan, D., Fuller, M., Maire, I., et al. (2008). Early neurodegeneration progresses independently of microglial activation by heparan sulfate in the brain of mucopolysaccharidosis IIIB mice. PLoS ONE *3*, e2296.

35. Zipp, F., and Aktas, O. (2006). The brain as a target of inflammation: common pathways link inflammatory and neurodegenerative diseases. Trends Neurosci. *29*, 518–527.

36. Block, M.L., and Hong, J.S. (2005). Microglia and inflammation-mediated neurodegeneration: multiple triggers with a common mechanism. Prog. Neurobiol. *76*, 77–98.

37. Parviainen, L., Dihanich, S., Anderson, G.W., Wong, A.M., Brooks, H.R., Abeti, R., Rezaie, P., Lalli, G., Pope, S., Heales, S.J., et al. (2017). Glial cells are functionally impaired in juvenile neuronal ceroid lipofuscinosis and detrimental to neurons. Acta Neuropathol. Commun. *5*, 74.

38. Li, H.H., Zhao, H.Z., Neufeld, E.F., Cai, Y., and Gómez-Pinilla, F. (2002). Attenuated plasticity in neurons and astrocytes in the mouse model of Sanfilippo syndrome type B. J. Neurosci. Res. *69*, 30–38.

39. Lee, S.T., Chu, K., Park, H.K., Jung, K.H., Kim, M., Lee, S.K., and Roh, J.K. (2008). New Concept of Neural Stem Cell Transplantation: Anti-inflammatory Role. Int. J. Stem Cells *1*, 36–42.

40. Casarosa, S., Bozzi, Y., and Conti, L. (2014). Neural stem cells: ready for therapeutic applications? Mol. Cell. Ther. *2*, 31.

41. Iacovino, M., Chong, D., Szatmari, I., Hartweck, L., Rux, D., Caprioli, A., Cleaver, O., and Kyba, M. (2011). HoxA3 is an apical regulator of haemogenic endothelium. Nat. Cell Biol. *13*, 72–78.

42. Li, H.H., Yu, W.H., Rozengurt, N., Zhao, H.Z., Lyons, K.M., Anagnostaras, S., Fanselow, M.S., Suzuki, K., Vanier, M.T., and Neufeld, E.F. (1999). Mouse model of Sanfilippo syndrome type B produced by targeted disruption of the gene encoding alpha-N-acetylglucosaminidase. Proc. Natl. Acad. Sci. USA *96*, 14505–14510.

43. Bible, E., Gupta, P., Hofmann, S.L., and Cooper, J.D. (2004). Regional and cellular neuropathology in the palmitoyl protein thioesterase-1 null mutant mouse model of infantile neuronal ceroid lipofuscinosis. Neurobiol. Dis. *16*, 346–359.

44. Kielar, C., Maddox, L., Bible, E., Pontikis, C.C., Macauley, S.L., Griffey, M.A., Wong, M., Sands, M.S., and Cooper, J.D. (2007). Successive neuron loss in the thalamus and cortex in a mouse model of infantile neuronal ceroid lipofuscinosis. Neurobiol. Dis. *25*, 150–162.

45. Macauley, S.L., Roberts, M.S., Wong, A.M., McSloy, F., Reddy, A.S., Cooper, J.D., and Sands, M.S. (2012). Synergistic effects of central nervous system-directed gene therapy and bone marrow transplantation in the murine model of infantile neuronal ceroid lipofuscinosis. Ann. Neurol. *71*, 797–804.

46. Griffey, M., Bible, E., Vogler, C., Levy, B., Gupta, P., Cooper, J., and Sands, M.S. (2004). Adeno-associated virus 2-mediated gene therapy decreases autofluorescent storage material and increases brain mass in a murine model of infantile neuronal ceroid lipofuscinosis. Neurobiol. Dis. *16*, 360–369.

47. Paxinos, G., and Franklin, K. (2001). The Mouse Brain in Stereotaxic Coordinates, Second edition (Academic Press).

OMTM, Volume *10*

Supplemental Information

Genetically Corrected iPSC-Derived Neural Stem Cell Grafts Deliver Enzyme Replacement to Affect CNS Disease in Sanfilippo B Mice

Don Clarke, Yewande Pearse, Shih-hsin Kan, Steven Q. Le, Valentina Sanghez, Jonathan D. Cooper, Patricia I. Dickson, and Michelina Iacovino

Supplementary Figure 1*. Biological Effect of NAGLU Treatment *in Vitro

Naglu$^{-/-}$ Neural Stem Cells (NSC) (A, B) or differentiated (C, D) cells treated for ten days with supernatant collected from Naglu overexpressing NSC (*N*-iNSCs) were compared to *Naglu*$^{+/+}$ untreated cells. (A) Nestin+ iNSCs, forward and side scatter (top) and LysoTracker staining (bottom). (B) FACS quantification: frequency of cell having bright LysoTracker signal (top) and mean LysoTracker signal (bottom). (C) Nestin-differentiated iNSCs, forward and side scatter (top) and LysoTracker staining (bottom). (D) FACS quantification: frequency cell having bright LysoTracker signal (top) and mean LysoTracker signal (bottom). Two-tailed *t*-test p-values .*$p < 0.05$, **$p < 0.01$, n = 3.

***Supplementary Figure 2*. Correction of Astrocytosis in *Naglu*$^{-/-}$ Mice Treated with Neural Stem Cells Overexpressing NAGLU (*N*-iNSCs) at Two Months**

(A) Schematic illustration intracerebroventricular (ICV, green) at bregma 1.54 mm; intraparenchymal (PAR, blue) at bregma -0.58 mm demonstrating regions of analysis (red squares), relative to the site of engraftment (ICV and PAR), including the motor cortex, striatum and ventral forebrain. (B) Representative bright-field images of half brain coronal sections of immunohistochemical staining of GFAP after *N*-iNSCs transplantation (-/- Treated) compared to unaffected heterozygous (Unaffected) and saline-injected controls (-/- Vehicle), scale bar = 1 mm. (C1-3) Representative immunohistological staining images for GFAP in higher magnification in the motor cortex (1), striatum (2) and ventral forebrain (3). Scale bar = 100 μm. Insert images taken

at higher magnification to highlight differences in morphology of GFAP-stained astrocytes, scale bar = 20 μm. Post-acquisition processing was applied to all images and included adjustments to brightness/contrast and Red Green Blue (RGB) curves using Adobe Photoshop CS6, to reduce improve visibility and consistency in colour tone. Area of GFAP immunoreactivity (μM^2) is summarized in the histograms. Values are shown as mean ± SEM (n = 3 mice per group), for p-values, see Table 1 and 2. *p < 0.05, **p < 0.01, ***p < 0.001.

Supplementary Figure 3. **Correction of Storage Material Accumulation in *Naglu*$^{-/-}$ mice Treated with Neural Stem Cells Overexpressing NAGLU (*N*-iNSCs) at Two Months**

(A) Schematic illustration intracerebroventricular (ICV, green) at bregma 1.54 mm; intraparenchymal (PAR, blue) at bregma -0.58 mm demonstrating regions of analysis (red squares), relative to the site of engraftment (ICV and PAR), including the motor cortex, striatum and ventral forebrain. (B) Representative bright-field images of half coronal sections immunostained for Lamp 1 in *Naglu*$^{-/-}$ mice (-/- Vehicle), heterozygotes (Unaffected) and *N*-iNSC transplanted (-/- Treated) mice. Scale bar = 1 mm. (C1-3) Representative bright-field images of the motor cortex (1), striatum (2) and ventral forebrain 9). Scale bar = 100 μm. Insert images taken at higher magnification to highlight cells with larger Lamp 1 staining lysosomes, scale bar = 20 μm. Post-acquisition processing was applied to all images and included adjustments to brightness/contrast and Red Green Blue (RGB) curves using Adobe Photoshop CS6, to reduce improve visibility and consistency in colour tone. Area of Lamp1 immunoreactivity (μM^2) is summarized in

the histograms showing differences between each treatment group. Values are shown as mean ± SEM ($n = 3$ mice per group) for p-values, see Table 1 and 2. *$p < 0.05$.

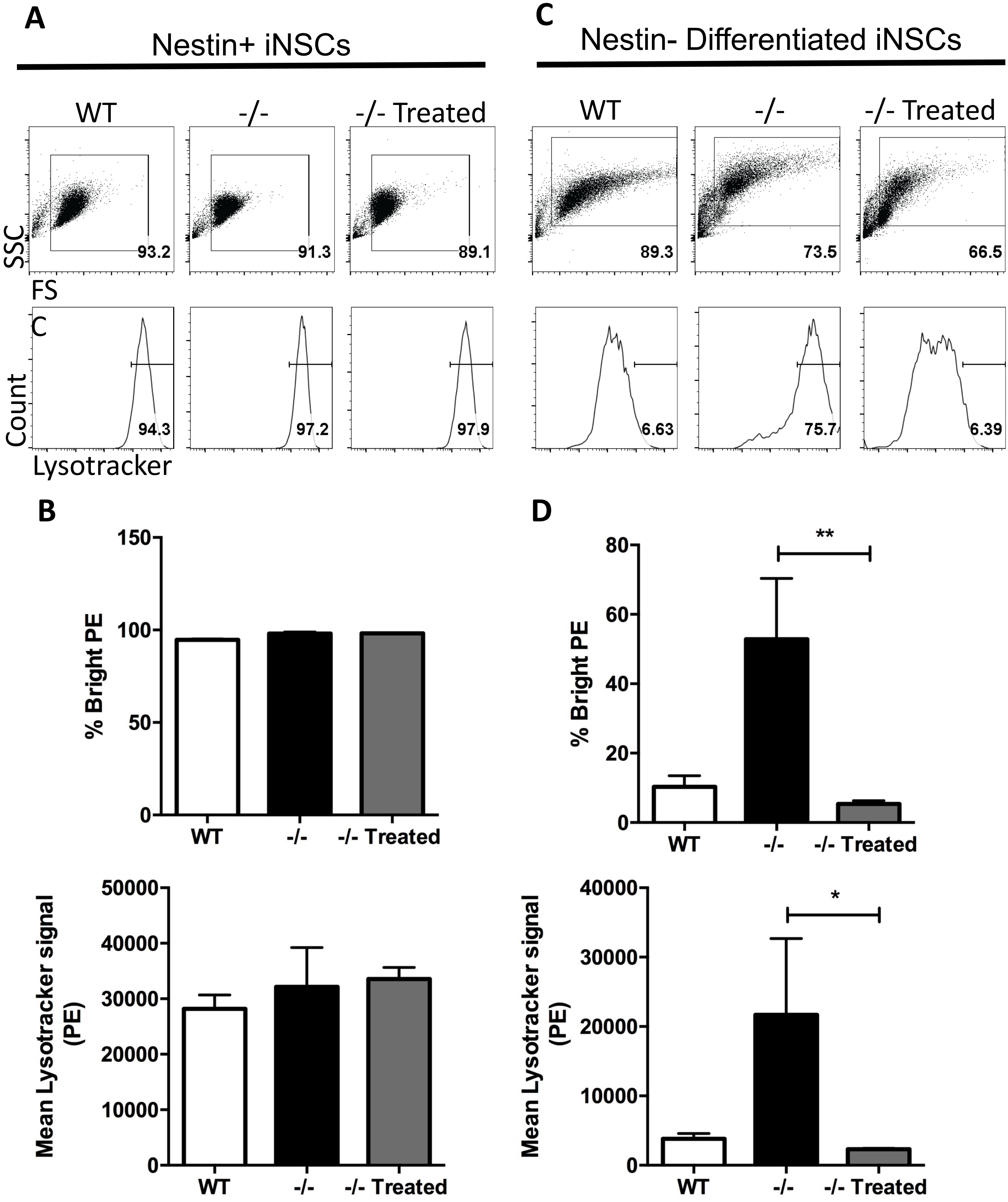

A
Nestin+ iNSCs
WT
-/-
-/- Treated
SSC
FS
93.2
91.3
89.1
Count
Lysotracker
94.3
97.2
97.9
C
Nestin- Differentiated iNSCs
89.3
73.5
66.5
6.63
75.7
6.39
B
% Bright PE
150
100
50
0
Mean Lysotracker signal (PE)
50000
40000
30000
20000
10000
D
80
60
40
20
**
*

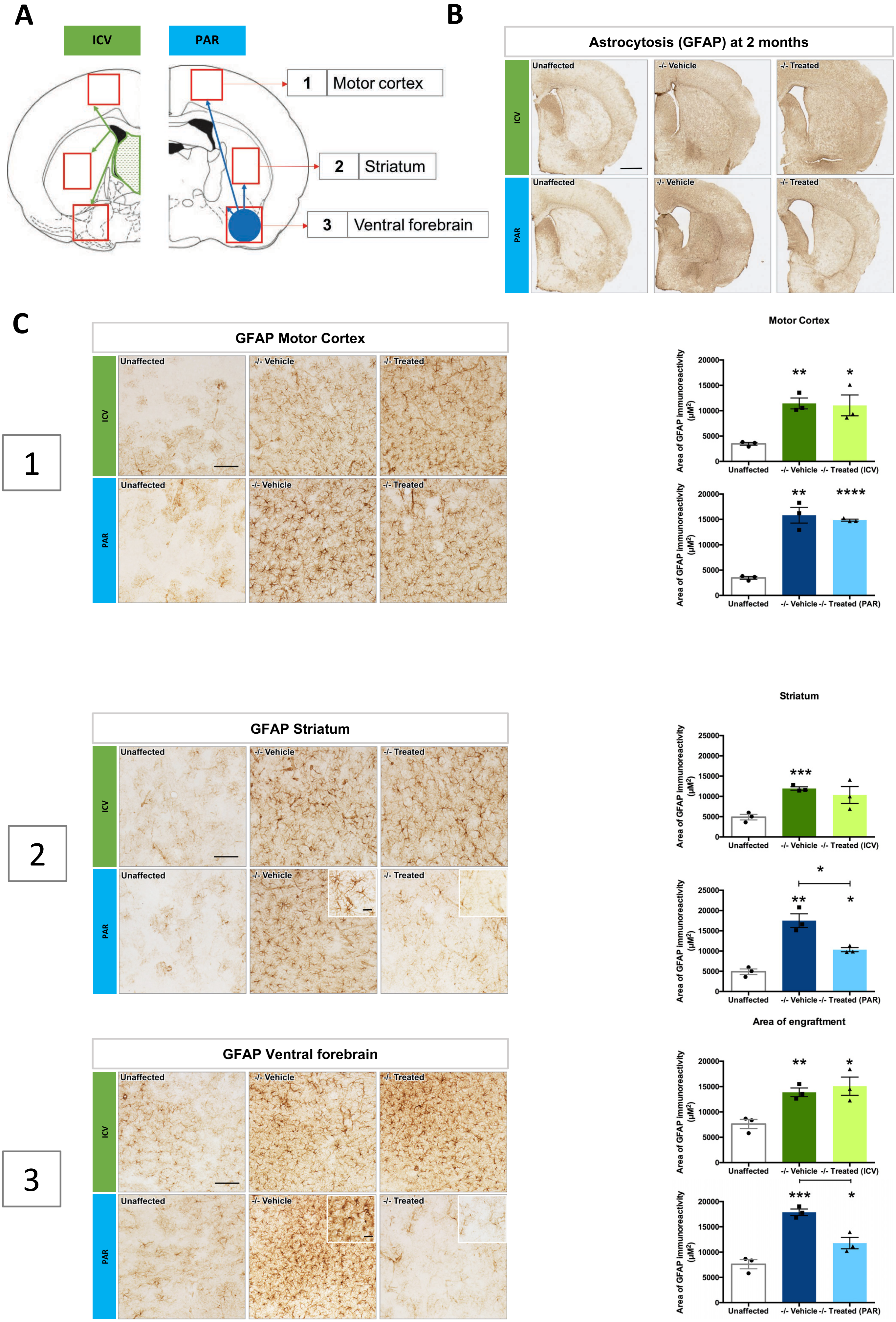
A
ICV
PAR
1 Motor cortex
2 Striatum
3 Ventral forebrain
B
Astrocytosis (GFAP) at 2 months
Unaffected
-/- Vehicle
-/- Treated
C
GFAP Motor Cortex
1
Motor Cortex
Area of GFAP immunoreactivity (μM²)
Unaffected
-/- Vehicle
-/- Treated (ICV)
-/- Treated (PAR)
GFAP Striatum
2
Striatum
GFAP Ventral forebrain
3
Area of engraftment

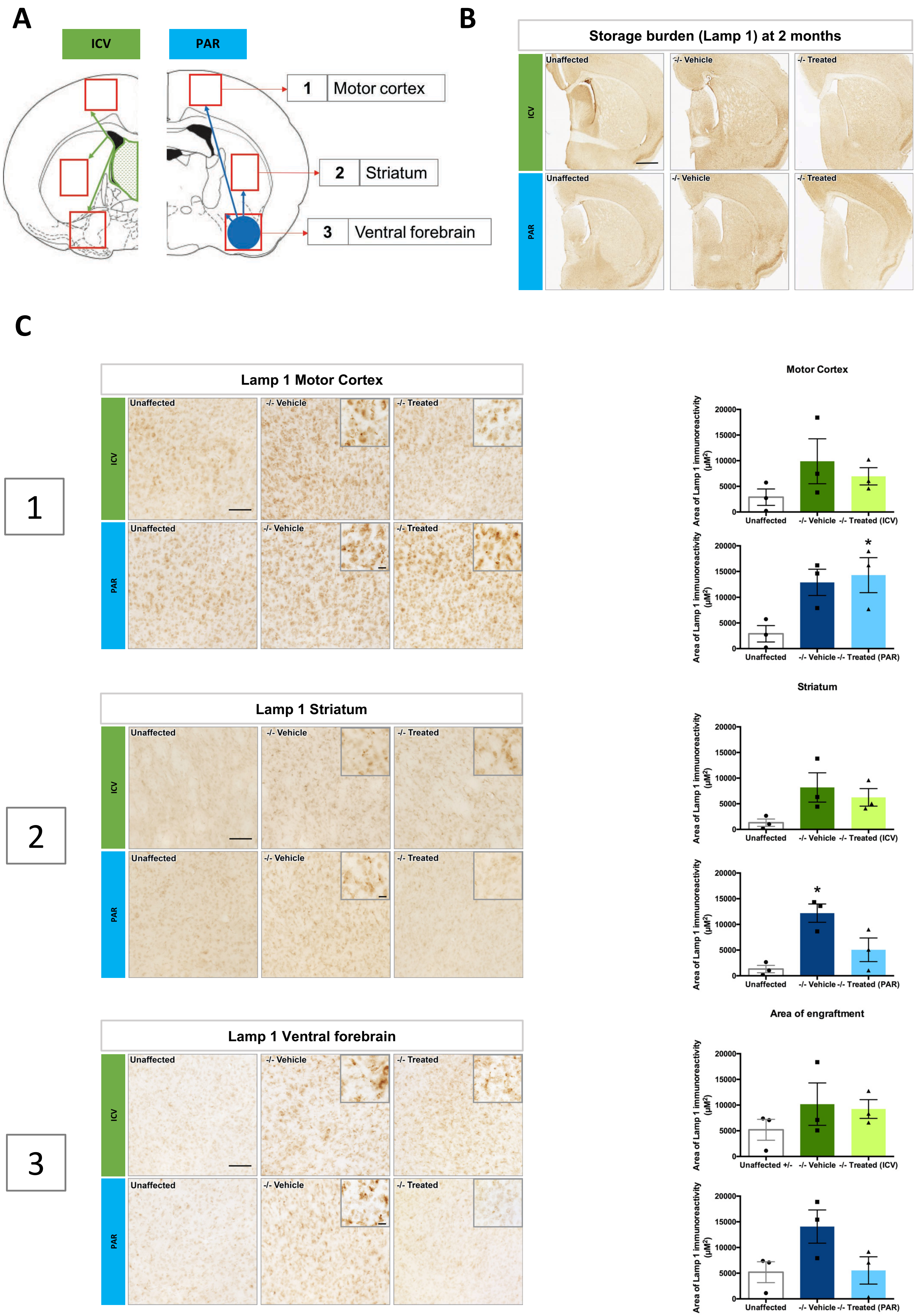

A
ICV
PAR
1 Motor cortex
2 Striatum
3 Ventral forebrain
B
Storage burden (Lamp 1) at 2 months
Unaffected
-/- Vehicle
-/- Treated
C
Lamp 1 Motor Cortex
Lamp 1 Striatum
Lamp 1 Ventral forebrain
Motor Cortex
Striatum
Area of engraftment
Area of Lamp 1 immunoreactivity (μM²)
Unaffected
-/- Vehicle
-/- Treated (ICV)
-/- Treated (PAR)
Unaffected +/-
